# Facile, environmentally benign and scalable approach to produce pristine few layers graphene suitable for preparing biocompatible polymer nanocomposites

**DOI:** 10.1038/s41598-018-28560-1

**Published:** 2018-07-25

**Authors:** Gejo George, Suja Bhargavan Sisupal, Teenu Tomy, Alaganandam Kumaran, Prabha Vadivelu, Vemparthan Suvekbala, Swaminathan Sivaram, Lakshminarayanan Ragupathy

**Affiliations:** 1 Corporate R&D Center, HLL Lifecare Limited, Akkulam, Sreekariam (P.O), Trivandrum, 695017 India; 2 CSIR-National Institute for Interdisciplinary Science and Technology, Industrial Estate (P.O), Pappanamcode, Trivandrum, 695019 India; 3 Polymers and Advanced Materials Laboratory, National Chemical Laboratory, Dr. Homi Bhabha Road, Pune, 411008 India

## Abstract

The success of developing graphene based biomaterials depends on its ease of synthesis, use of environmentally benign methods and low toxicity of the chemicals involved as well as biocompatibility of the final products/devices. We report, herein, a simple, scalable and safe method to produce defect free few layers graphene using naturally available phenolics i.e. curcumin/tetrahydrocurcumin/quercetin, as solid-phase exfoliating agents with a productivity of ∼45 g/batch (D/G ≤ 0.54 and D/D′ ≤ 1.23). The production method can also be employed in liquid-phase using a ball mill (20 g/batch, D/G ≤ 0.23 and D/D′ ≤ 1.12) and a sand grinder (10 g/batch, D/G ≤ 0.11 and D/D∼ ≤ 0.78). The combined effect of π-π interaction and charge transfer (from curcumin to graphene) is postulated to be the driving force for efficient exfoliation of graphite. The yielded graphene was mixed with the natural rubber (NR) latex to produce thin film nanocomposites, which show superior tensile strength with low modulus and no loss of % elongation at break. *In-vitro* and *in-vivo* investigations demonstrate that the prepared nanocomposite is biocompatible. This approach could be useful for the production of materials suitable in products (gloves/condoms/catheters), which come in contact with body parts/body fluids.

## Introduction

Graphene has the ability to revolutionize many research fields including energy technology, sensors, composites and biomaterials^1–6^ because of its unique and outstanding physical properties, namely, stretchability (20% of its initial length), high modulus (~1100 GPa), extraordinary electrical conductivity (mobility of charge carriers 200,000 cm^2^ V^−1^ s^−1^), huge surface area (2630 m^2^/g) and superior thermal conductivity (~5000 W/mK)^7–11^. Graphene based materials (GBMs) for biomedical applications such as biosensing, bioimaging^12,13^, drug delivery^12,14^, cancer photothermal therapy^12,15^, and antibacterial materials, have been widely investigated. The advantages of GBMs are (i) enhanced mechanical/electrical/thermal (conductivity and stability) properties (ii) improvement of cellular attachment and growth at GBMs surface and (iii) capability of loading and delivering high amounts of drugs. The main concern in using GBMs in biomedical field is the biocompatibility, which depends on (a) physico-chemical properties of GBMs (b) raw materials used and (c) production methods employed^12^. Therefore, the reported investigations on biological effects of GBMs often show contradictory or inconclusive results. At the same time, when graphene is incorporated into a polymer matrix, the toxicity of the filler is reduced. This is due to minimization of direct biological interactions with the encapsulated materials. Most of the studies employ graphene oxide (GO)/reduced graphene oxide(rGO)/functinalized GO for the preparation of the biocompatible naocomposites. However, use of few layers pristine graphene based biocompatible composite materials has not been studied widely^16^.

The commercial utility of biocompatible GBMs depends on the choice of graphene (GO/rGO/functionalized GO/few to multiple layer graphene), its production method and the technique used to incorporate graphene into a polymer matrix, the scalability of these methods as well as biocompatibility of the prepared composites. Two-dimensional defect free graphenes are prepared using chemical vapour deposition and SiC methods and are suitable for electronic applications. However, for less demanding applications several top-down mechano-chemical methods can be used which include, chemical exfoliation using Hummers method, sonication, solvent- and/or surfactant-facilitated liquid-phase exfoliation, electrochemical exfoliation, shear exfoliation and wet as well as dry ball milling with suitable organic chemical additives^17–25^. Among these methods, shear exfoliation and ball milling with graphite exfoliating agents are relatively simpler to practice, scalable, more economical and result in graphenes consisting of few layers with fewer defects^25–27^. More recently, Paton *et al*. reported a method to produce defect free few layer graphene by liquid-phase shear exfoliation. The exfoliation was performed in a medium consisting of N-methyl-2-pyrrolidone, aqueous solutions of sodium cholate and polyvinyl alcohol and a production rate of 5.3 gh^−1^ was demonstrated^28^.

For biomedical applications, one has to consider the adverse effects of chemicals and solvents used for exfoliation. Many of the currently reported chemicals and diluents for exfoliation of graphite do not meet the safety requirements needed for applications of graphenes where it comes into contact with body parts or body fluids. Only limited studies are available on the use of less/non- toxic exfoliating chemicals i.e. cyrene/gum arabic/pluronic/tetronics (Table 1). However, the production rate in these cases was limited to 0.06 gh^−1^. A careful search of the literature (Table S4) shows that currently no method exist for graphite exfoliation that employs a combination of low toxicity organic compounds, is simple, has reasonable production rate and gives defect free few layers graphene (Table S4).

**Table 1:** Safe exfoliating agents employed in the literature and its performance (i.e. production rate, defect level and demonstrated application).

Ref.	Exfoliation agents	Production method	Reported no. of graphene layers	Graphene production rate (yield) in g/h	Raman D/G	Is application demonstrated?
^69^	Gum arabic	Sonication	5–20	~6 × 10^−3^	~0.25 (633 nm)	No
^24^	Cyrene	Sonication and centrifuging	Mono to few layer graphene (<10)	0.06	0.20	No
^70^	Pluronic/tetronics	Sonication	1 to ∼10 layers	1.1 × 10^−3^	0.9 (514 nm)	No
Current work	Curcumin/tetrahydro curcumin/quercetin	Planetary ball milling and Sand Grinder	Bilayers to few and multi layers	45/20/10	≤ 0.54/ ≤ 0.23/ ≤ 0.11 (514 nm)	Produced biocompatible thin film (NR latex) nanocomposite

**Figure 1:**
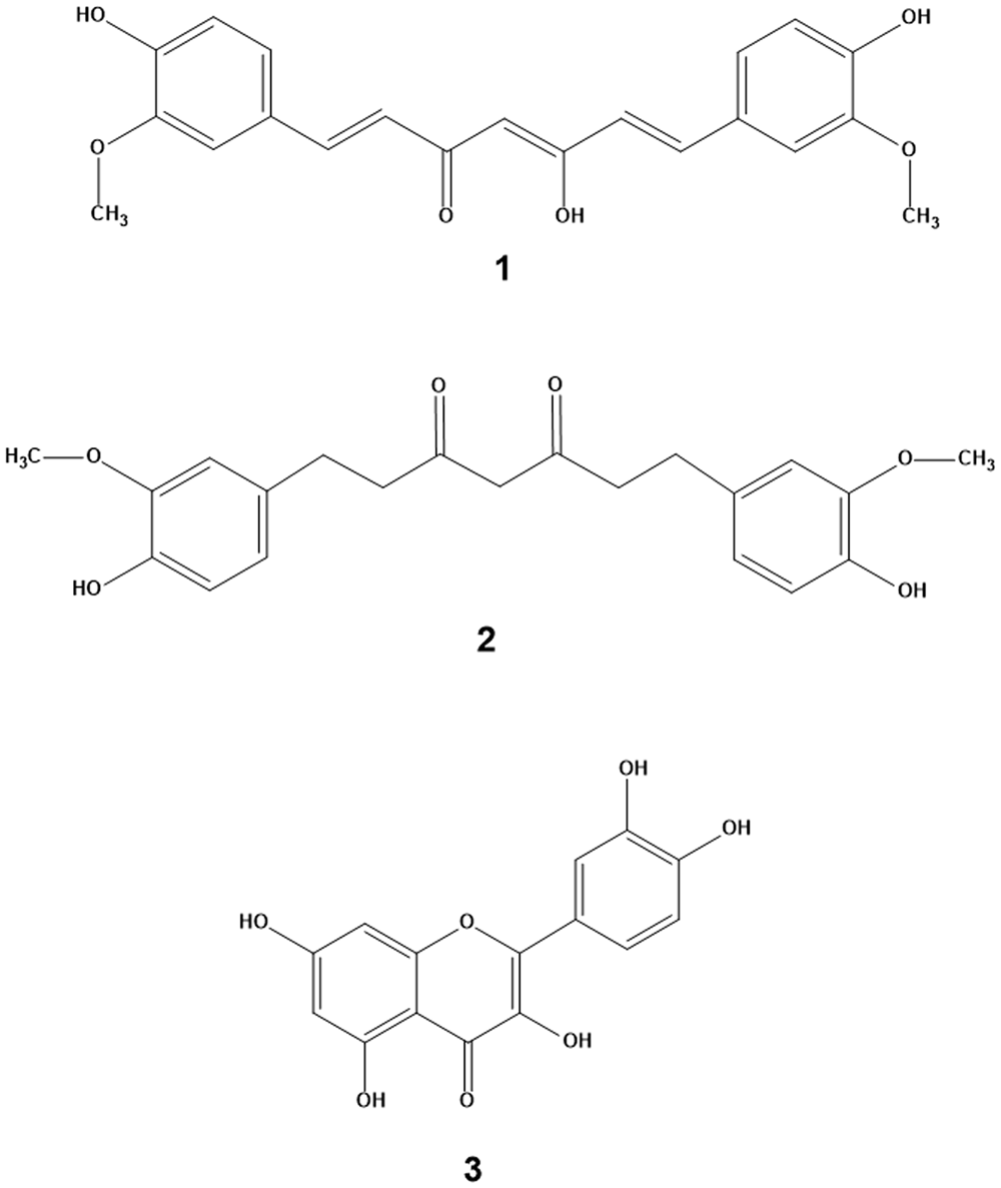
Chemical structure of (1) curcumin (2) tetrahydrocurcumin and (3) quercetin.

In this paper, we report a simple and potentially scalable method of preparing defect free few layers graphene using naturally available polyphenols such as curcumin, tetrahydrocurcumin and quercetin. Curcumin {Fig. 1(1)} is a naturally occurring diphenol and is the principal constituent of turmeric and ginger and has been widely explored for various pharmacological properties^29–31^. Tetrahydrocurcumin **{**Fig. 1(2)} is one of the product of bacterial and intestinal metabolism of curcumin^32^ and has been reported to possess a stronger antioxidant property than curcumin^33^. Quercetin {Fig. 1(3)} is a plant derived flavonoid and found in fruits, vegetables, leaves and grains and is reported to be one of the most potent antioxidants along with other therapeutic properties like anti-inflammatory, anticancer etc^34^. All the above compounds are generally regarded as safe (GRAS). Additionally, we demonstrate the utility of such few layer graphene for the preparation of graphene-NR latex nanocomposite thin films with improved mechanical properties. Such thin film nanocomposites were also found to be biocompatible. This study opens up new ways for making biocompatible nanocomposites with polymers and their application in commercially important medical products with desirable properties and product safety.

## Results and Discussion

### Exfoliation of graphite with naturally available Curcuminoids

In view of the inherent chemical safety of the curcuminoid classes of compounds, we explored their ability to exfoliate graphite using wet and dry grinding methods. We reasoned that the electron rich aromatic groups as well as acidic protons in such molecules may result in favorable interaction with delaminated layers of graphene. To the best of our knowledge, aromatic phenols have not been thus far demonstrated as useful chemicals for exfoliating graphite. Different weight ratios of graphite and Curcumin or Tetrahydrocurcumin or Quercetin i.e. 1:0.5, 1:1, 1:2 and 1:3, were dry ground using a planetary ball mill. With Quercetin, only 1:3 ratio was employed. Grinding was performed using a Retsch PM 400 planetry Ball mill using 50 balls having 10 mm dia. A 75 mL sample jar was used with a calculated free volume of 60%. Based on this, the rate of production of few layer graphene is estimated to be around 45 g per batch under these conditions.

**Figure 2:**
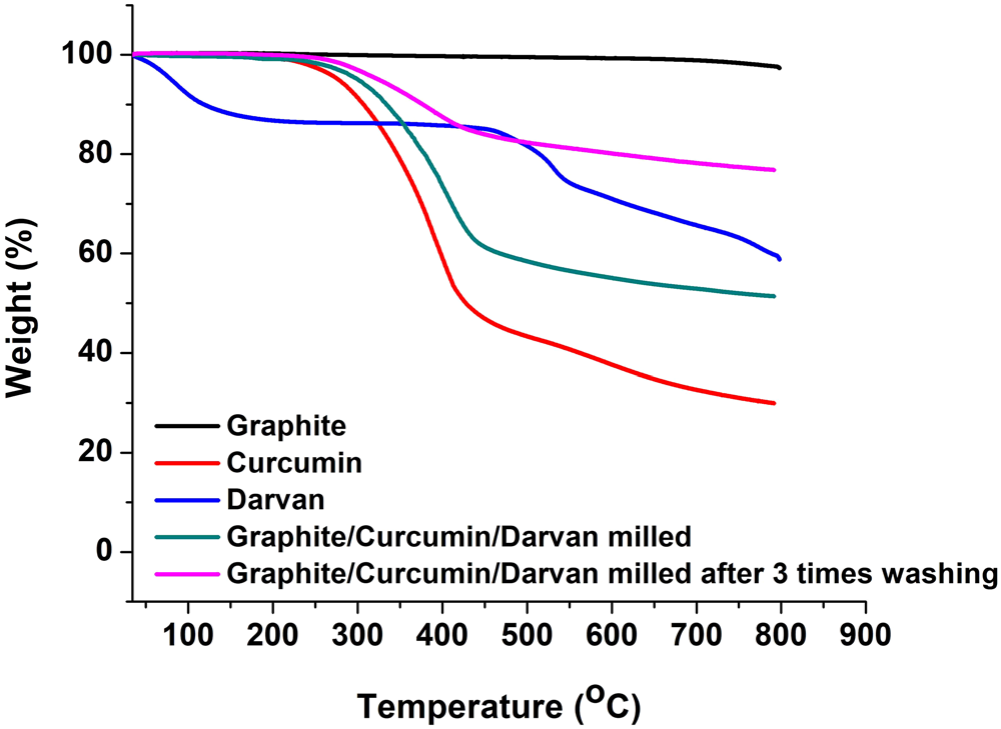
Thermogravimetric analysis of ball milled Graphite:Curcumin:Darvan samples.

A surfactant, sodium polynaphthalenesulphonate (Darvan) was added to improve the dispersion of exfoliated graphite in water. Control experiments were performed to show that Darvan (at 12.5 wt% of graphite) alone is incapable of exfoliating graphite. After grinding, the samples were washed thoroughly with a 50:50 wt% acetone and water mixture (to remove excess curcumin, and tetrahydrocurcumin) or with 50:50 wt% methanol and water mixture (to remove quercetin). A typical ball milled sample (Graphite:Curcumin:Darvan), before and after washing with acetone water mixture was examined by TGA (Fig. 2). No weight loss is observed when graphite is heated to 800 °C in nitrogen. With the Graphite:Curcumin:Darvan sample weight loss is observed at 360 °C which corresponds to the loss of curcumin. A char residue of about 30 wt% is observed that is attributed to the presence of two phenyl rings in curcumin. A similar observation has been reported by Luo *et al*. in case of cellulose-curcumin composites^35^. TGA shows the presence of about 24 wt% exfoliated graphite, which corresponds to the amount of graphite originally used. There are no other weight loss taking place being observed, indicating that no oxidative defects have been created around the graphite flakes during grinding^21,22^. Furthermore, TGA indicates that the sample after washing with 50:50 wt% water and acetone mixture (10 mL/g × 3 times) is free of 80% curcumin.

**Figure 3:**
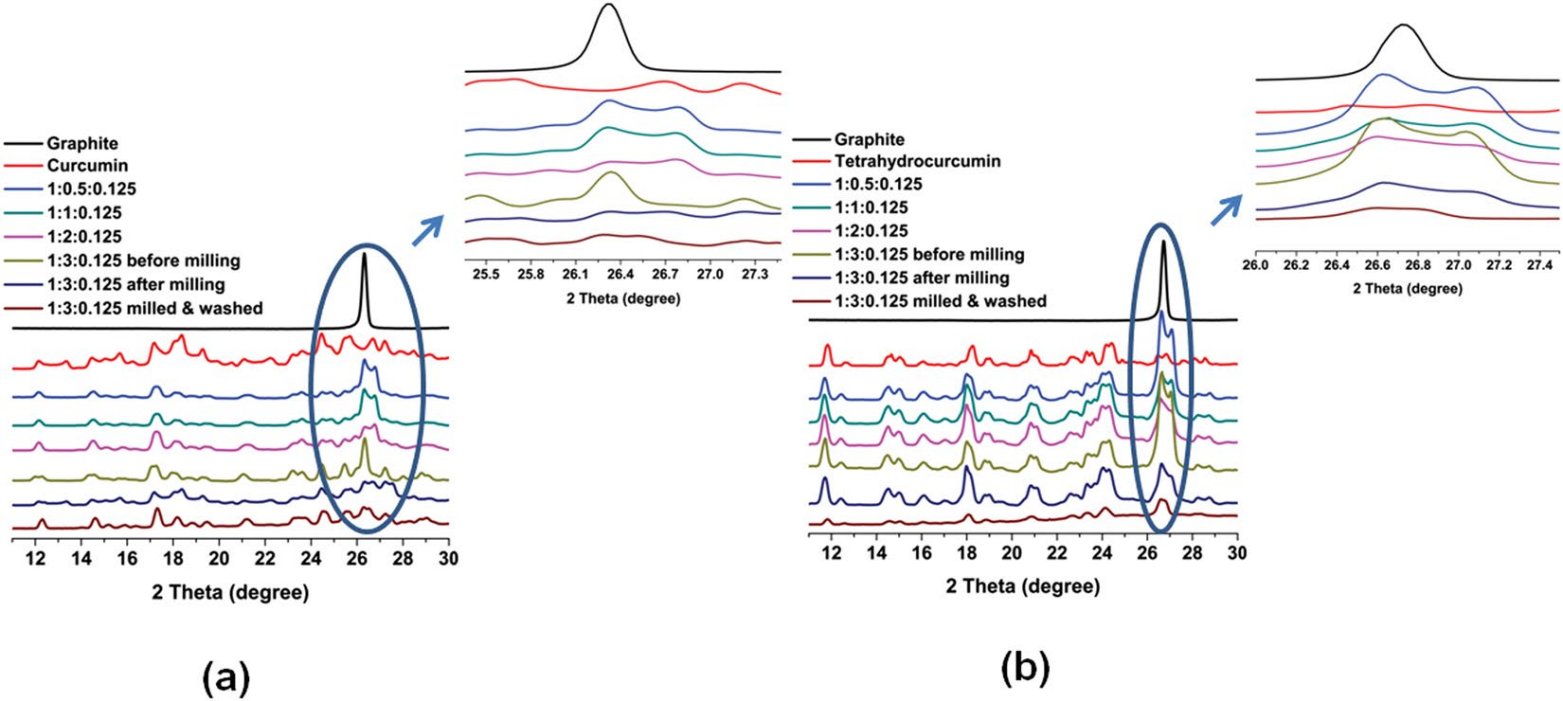
Normalized XRD of exfoliated graphite samples produced using (**a**) Curcumin (the given ratios represent Graphite:Curcumin:Darvan, respectively) and (**b**) Tetrahydrocurcumin (the ratios correspond to Graphite:TetrahydroCurcumin:Darvan, respectively) as the exfoliating agent.

The normalized X-Ray diffractograms of exfoliated graphite before and after ball milling as well as after washing, using curcumin and tetrahydrocurcumin as the exfoliating agents are shown in Fig. 3a,b, respectively. It is observed that the sharp graphitic (002) reflection at around 27° decreases with increasing amount of curcumin and tetrahydrocurcumin and reaches a minimum at the Graphite:Curcumin:Darvan or Graphite:TetrahydroCurcumin:Darvan weight ratio of 1:3:0.125. The normalized XRD of ball milled Graphite:Quercetin:Darvan in the weight ratio of 1:3:0.125 also exhibits a similar behavior (Figure S1) and suggesting that all the three investigated molecules are effective in exfoliating graphite.

**Figure 4:**
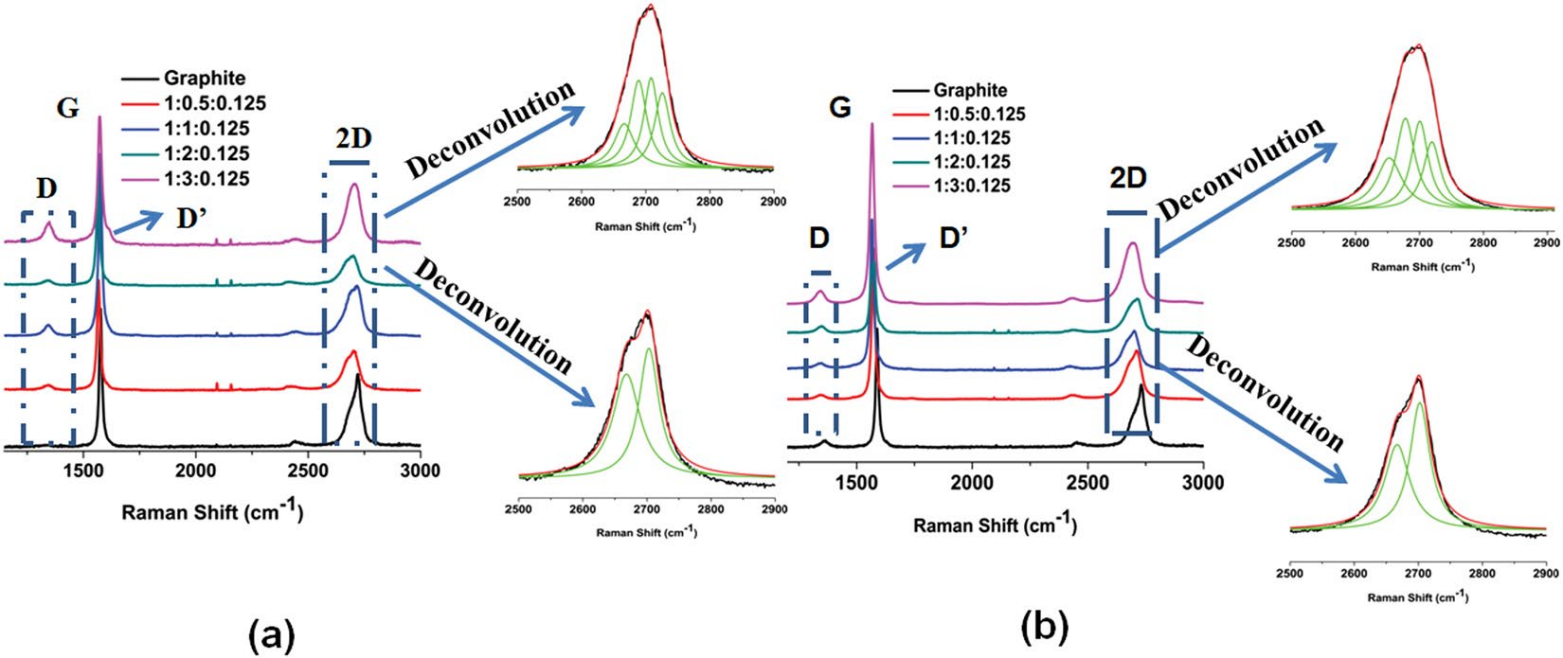
Raman spectra of exfoliated graphite samples produced using (**a**) Curcumin (the ratios denote Graphite:Curcumin:Darvan, respectively) and (**b**) Tetrahydrocurcumin (the ratios mean to Graphite:TetrahydroCurcumin:Darvan, respectively) as the exfoliating agent.

The above-mentioned samples were subjected to Raman spectroscopy (Fig. 4a,b and Figure S2). Graphite and graphene exhibit characteristic G and 2D peaks at 1580 and 2700 cm^−1^, respectively. In addition, graphene show extra Raman peaks at around 1345 cm^−1^ (D band) and 1626 cm^−1^ (D′ band). D′ appears as a shoulder on the G band and is characteristic of few layers graphenes^21,36–40^. Graphite displays sharp 2D band at around 2700 cm^−1^, while, for few layer graphene the 2D band is broad and is shifted to a lower wavelength. The deconvolution of 2D band in case of Graphite:Curcumin:Darvan and Graphite:TetrahydroCurcumin:Darvan samples (1:3.0:0.125 wt%) leads to four Lorentzian peaks, which are distinctive of bi-layer graphene^21,38,39^. For other ratios of Graphite:Curcumin:Darvan and Graphite:TetrahydroCurcumin:Darvan samples, the deconvolution of the 2D Raman peak shape results in two Lorentizian peaks indicative of 5 layers graphene^21,38^. In the case of Graphite:Quercetin:Darvan sample, the 2D band shape is observed to be different from that of the samples prepared with curcumin or tetrahydrocurcumin and is characteristic of a 10 layer graphene^21,36,38,39,41^.

The intensity ratio of D and D′ bands (I_D_/I_D′_) was used to estimate the nature of defects (e.g. sp^3^, vacancy and edge)^21,28,42^. For sp^3^ type defects I_D_/I_D′_ is ∼13, which decreases to ∼7 for vacancy-like defects. Boundary like/edge like defects exhibits the smallest value of I_D_/I_D′_^21,28,42^. The I_D_/I_D′_ values are very low, between 0.74 to 1.23 for Graphite:Curcumin:Darvan and 0.85–1.23 for Graphite:TetrahydroCurcumin:Darvan. The I_D_/I_G_ ratio is proportional to inverse nano-sheet length and found to be 0.40–0.54 for Graphite:Curcumin:Darvan, 0.21–0.35 for Graphite:TetrahydroCurcumin:Darvan and 0.10–0.21 for Graphite:Quercetin:Darvan. These results indicate that the exfoliated graphenes are high quality few layer graphenes, which possess only edge or boundary like defects and no new vacancy or basal plane defects introduced during ball milling. Thus, it can be concluded that with the use of appropriate exfoliating agent (chemical nature and amount), it is possible to prepare graphenes with two to ten layers.

**Figure 5:**
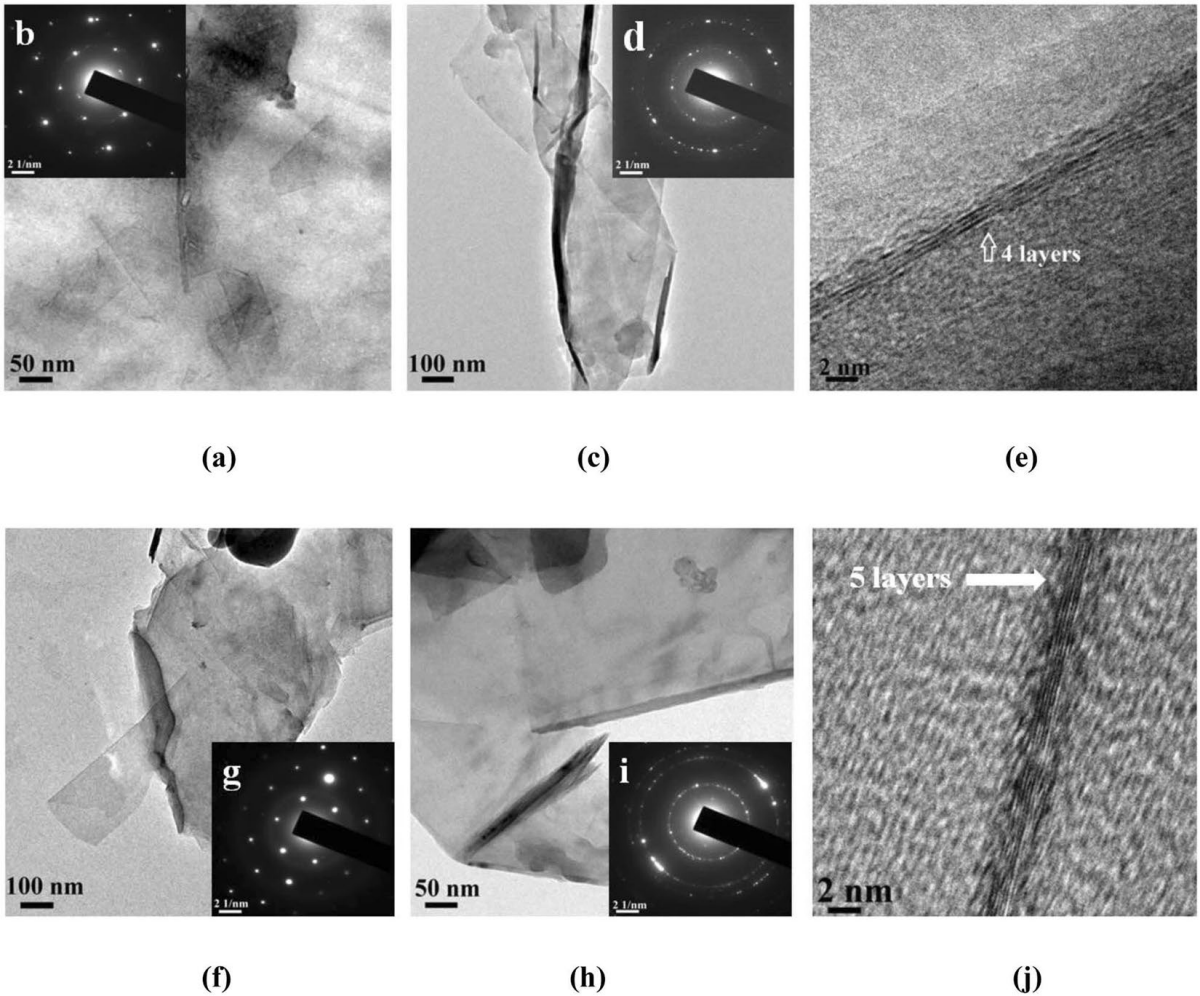
TEM images of (**a**) and (**c**) Graphite:Curcumin:Darvan (1:3:0.125) dispersion in water, (**b**) & (**d**) SAED patterns of the above said images, (**e**) Graphene fringes observed in the image (**c**). TEM images of (**f**) & (**h**) Graphite:TetrahydroCurcumin:Darvan (1:3:0.125) dispersion in water, (**g**) & (**i**) SAED patterns of the above said images and (**j**) Graphene fringes observed in the image (**h**).

Transmission electron microscopy of exfoliated graphite exhibit sheet like structure when curcumin and tetrahydrocurcumin are used as exfoliating agents (Fig. 5a,c and f,h). SAED pattern of exfoliated graphite prepared using curcumin and tetrahydrocurcumin shows a single set of symmetric six-fold diffraction spots (Fig. 5b and g). The outer group of diffraction spots from equivalent planes (1−210) has higher intensity than the inner set (1−100). This is a typical characteristic feature of bilayer graphene^43–45^. SAED pattern of the sheet like samples shown in Fig. 5d,i indicates that most regions of the graphene film have a hexagonal diffraction pattern attesting the crystalline nature of the film. Multiple sets of diffraction in the SAED pattern is evidence for the existence of few layer graphene^43–45^. In addition, graphene fringes corresponding to 4 and 5 layers are also clearly visible (Fig. 5e,j).

We also explored a wet grinding method using a mixture of acetone and water, with acetone content varying between 0 and 100% by weight for exfoliating graphite using curcumin as the exfoliating agent (1:3:0.125 wt. ratio). XRD analysis confirms that exfoliation of graphite upon wet grinding also (Figure S7). The Raman spectroscopy shows that the bilayer graphene (2D band deconvolutes into 4 Lorentizian peaks) is observed only when a 50:50 wt% acetone:water mixture was used. In other ratios of solvent, only 5 layer graphenes (deconvolutes as 2 Lorentizian peaks) are observed (Figure S8). TEM images (Figure S9) show a sheet like structure along with multiple sets of diffraction in the SAED, indicating the formation of few layer graphene^43–45^.

Sand grinder is a common milling technique used for paint and latex processing to make uniform dispersions. We also attempted the use of a sand grinder for exfoliating graphite. A 30 wt% Graphite:Curcumin:Darvan (1:3:0.125 wt. ratio) in water was fed into a sand mill and ground for 1 h (production rate 10 g per batch). The resulting product was analyzed using XRD, Raman spectroscopy and TEM (including SAED) (Figure S10–12). The results show that commonly used industrial equipment such as a sand mill is also quite efficient in exfoliating graphite to few layer graphene.

### Mechanism of graphite exfoliation using curcumin

To better understand the mechanism of exfoliation by curcumin, we undertook a computational modeling study using both Density Functional Theory (DFT) and semi empirical methods. The nature of interactions of adsorbed molecule on graphene is often governed by weak dispersive interactions between molecules^46^. Hence the dispersion-corrected DFT method (B97-D)^47^ implemented in G09^48^ and PM7^49^ method augmented in MOPAC^50^ was used to calculate the adsorption energy (E_ads_) of curcumin on graphene surface. E_ads_ has been calculated by subtracting the energy of the isolated graphene (E_G_) and curcumin (E_C_) molecules from the total energy of graphene and curcumin complex (E_G-C_) as given in (1).

1 $${{\rm{E}}}_{{\rm{ads}}}={{\rm{E}}}_{{\rm{G}} \mbox{-} {\rm{C}}}-{{\rm{E}}}_{{\rm{G}}}-{{\rm{E}}}_{{\rm{C}}}$$

**Figure 6:**
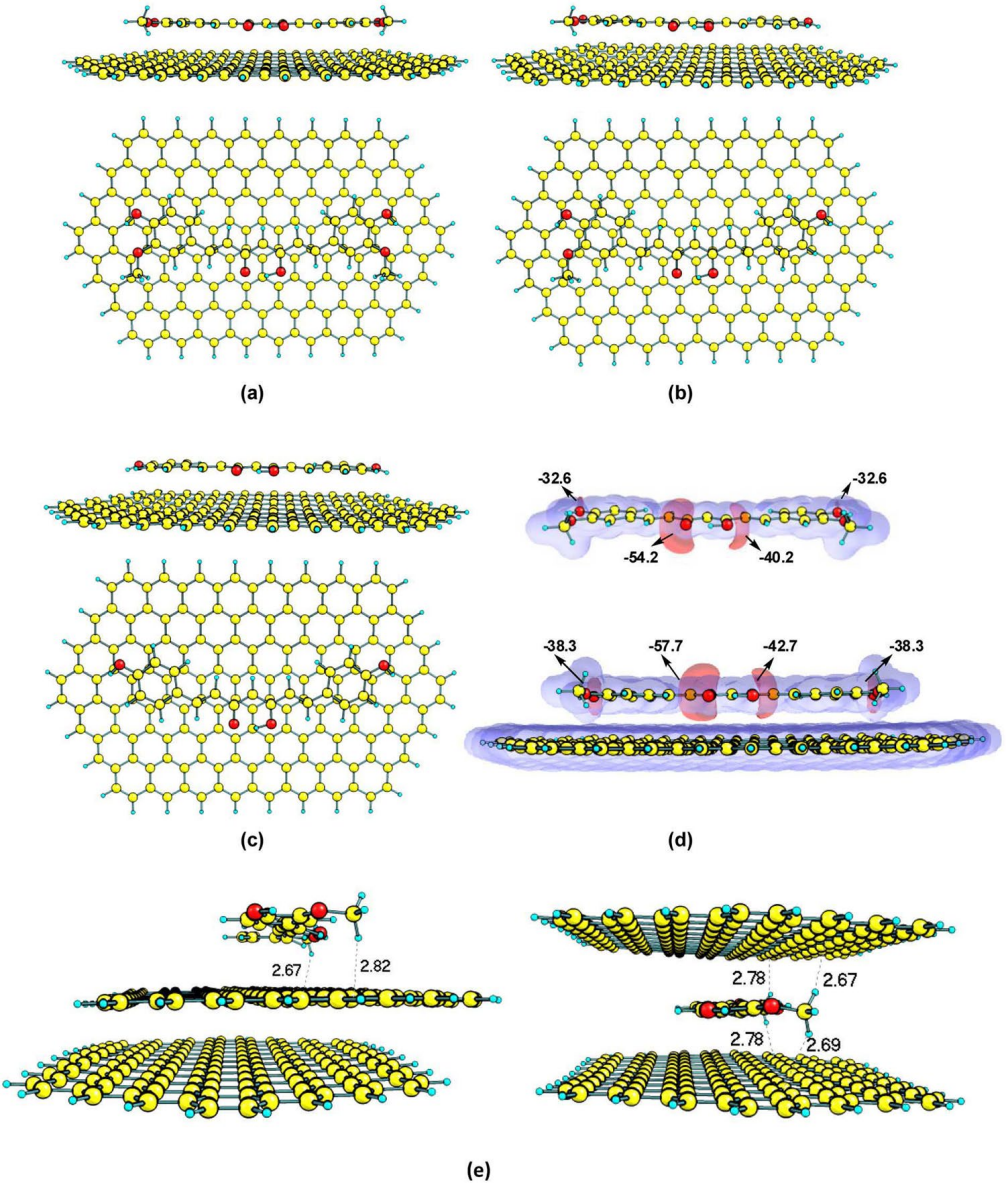
Views of the optimized structures of curcumin adsorbed on the C_170_H_32_ graphene sheet. (**a**) Curcumin, (**b**) Demethoxycurcumin, (**c**) Bisdemethoxycurcumin. (**d**) Molecular electrostatic potential map and the V_min_ values of lone pair electrons (kcal/mol) of free curcumin and the absorbed one on the C_170_H_32_ graphene sheet. (**e**) Curcumin on the top and in-between the two C_160_H_32_ graphene sheets, distances are shown in Å.

Initially, three different isomers of curcumin in the keto-enolic form were optimized using the B97-D method (Figure S13). As previously observed^51^, the keto and enolic form of curcumin isomers exhibit equal stability and are more stable than the di-keto form by c.a 5.2 kcal/mol. Hence, the more stable keto-enolic isomer of curcumin (one among the three isomers) was considered (Fig. 6a) for computational studies. The adsorption energy of dimethoxy (Fig. 6b) and bisdemethoxycurcumin (Fig. 6c) was also estimated on the graphene surface since demethoxy and bisdemethoxy curcumins exist along with curcumin in the ratio of 0.17:0.03:0.77^52^. A graphene sheet consisting of 160 carbons and 32 peripheral hydrogens (C_160_H_32_) was initially modeled at B97-D method. The E_ads_ of curcumin on C_160_H_32_ is found to be −60.0 kcal/mol, which is higher than the E_ads_ of demethoxy and bisdemethoxy curcumins by c.a. 4–7 kcal/mol (Table 2). The higher E_ads_ of the curcumin compared to the demethoxy derivative is most likely due to the presence of two -OCH_3_ groups, wherein, the shortest distance from the C_160_H_32_ graphene surface to curcumin is located for the hydrogen atom of the -OCH_3_ (2.60 Å). Furthermore, the E_ads_ of curcumin is appreciably larger than the reported E_ads_ of melamine (−24.3 kcal/mol) or melamine dimer (−44.6 kcal/mol) on the graphene surface^21^. This can be attributed to the longer pi-conjugation and higher π-π stacking interaction of adsorbed curcumin compared to melamine. The effect of the graphene model on E_ads_ is investigated by taking two other graphene sheets containing zigzag and armchair edges viz. C_168_H_36_ and C_156_H_36_ (see SI Section S6 for the optimized geometries). The E_ads_ of curcumin on C_168_H_36_ and C_156_H_36_ (Table 2, E_ads_ = −59.7 kcal/mol and −58.5 kcal/mol) are in agreement with that of C_160_H_32_. This shows that the choice of graphene model has no effect on E_ads_ of curcumin. In order to investigate the charge transfer during graphene-curcumin complex formation, the Natural Bond Orbital analysis was performed. The natural charge of all the atoms of graphene (C_160_H_32_, C_168_H_36_ and C_156_H_36_) during the adsorption of curcumin is found to be −0.004 |e|, −0.02 |e| and −0.01 |e|, respectively. The negative charge value on the graphene sheet indicates that the transfer of charge occurs from curcumin to graphene. The molecular electrostatic potential was also investigated in the case of C_160_H_32_ and curcumin (Fig. 6d), in which the negative potential is mainly concentrated on the oxygen atoms of curcumin and this could be presumably transferred to graphene surface during curcumin adsorption. The E_ads_ of curcumin on C_160_H_32_, C_168_H_36_ and C_156_H_36_ graphene models were also calculated using PM7 method. The E_ads_ calculated using the PM7 method agrees well with the B97-D results (a deviation of 1–4 kcal/mol). This suggests that the PM7 method is of comparable accuracy with the computationally demanding B97-D method to describe the weak dispersive interaction between curcumin and graphene.

**Table 2:** Adsorption energies (E_ads_) for curcumin and demethoxy curcumin derivatives on the graphene sheet calculated at B97D and PM7 levels.

Exfoliating agent	Graphene sheet	E_ads_ in kcal/mol (B97-D)	E_ads_ in kcal/mol (PM7)
Curcumin	C_160_H_32_	−60.0	−60.1
Demethoxy curcumin	C_160_H_32_	−56.3	−54.7
Bisdemethoxy curcumin	C_160_H_32_	−52.6	−49.5
Curcumin	C_168_H_36_	−59.7	−56.0
Curcumin	C_156_H_36_	−58.5	−59.4

In order to investigate the exfoliation mechanism, PM7 calculations were performed with two layers C_160_H_32_ graphene sheets, wherein, the curcumin is allowed to interact either on the top or in-between the two C_160_H_32_ graphene sheets. As shown in Fig. 6e, during the interaction of curcumin on the top layer of C_160_H_32_ sheets, the bottom layer smoothly slides over. This is probably due to the adsorption of curcumin on the top layer accompanied by charge transfer, which renders π-π interaction between C_160_H_32_ graphene sheets very weak. The interaction of curcumin between the two C_160_H_32_ graphene sheets keeps the layers aligned at an equal distance. However, subsequent adsorption of curcumin withC_160_H_32_ sheets will lead to delamination of graphene layers due to the stronger interaction of curcumin on both the layers. Hence, PM7 results support the hypothesis that the exfoliation of graphite is due to the strong adsorption of curcumin on the delaminated graphene surface and consequent weakening of the π-π interaction between the graphene layers.

### Few layer graphene-NR thin film nanocomposite

Due to the outstanding properties of graphene, graphene-elastomer nanocomposites have been extensively studied and reviewed^53–58^. However, only few reports are available on NR latex graphene nanocomposites. In most of these studies, graphene oxide (GO) was used and dispersed into NR latex followed by *in-situ* reduction, coagulation and compounding on a two roll mill^59–66^. Under these conditions, the unreduced functionalities remaining in rGO increase the curing kinetics, leading to scorching and consequent increase in the degree of cross-linking. Consequently such composites show higher modulus and reduced elongation at break^65,67^. Recently, we have reported a method to incorporate few layers graphene (prepared using melamine as an exfoliating agent) into NR latex and produce a thin film nano-composite, which show significant improvement in tensile strength with only modest increment in modulus^68^.

We, therefore, extended the study to few layers graphene-NR naocomposite prepared using curcumin as the exfoliating agent. Stable aqueous dispersion of few layer graphenes (Graphite:Curcumin:Darvan, 1:3:0.125, dry ground) was prepared by probe sonication. A laboratory model dipping machine was used to prepare the nanocomposite thin films followed by curing in hot air oven for 45 min. at 80 °C. To investigate the effect of graphene content in the NR latex, different concentrations of few layer graphene (0.3, 0.7, 1.5, 3 and 5 phr) were incorporated into the NR latex. For purposes of comparison, (a) NR latex (without graphene), (b) curcumin (milled with Darvan), (c) graphite (milled graphite without an exfoliating agent) and (d) three different carbon blacks (high abrasion furnace/semi-reinforced/super abrasion furnace) composite thin films were also prepared.

Tensile strength, tensile modulus and elongation at break of the produced few layer graphene-NR thin film nanocomposites are given in Table 3. A 16, 23 and 36% increase in tensile strength, respectively, was observed with 0.3, 0.7 and 1.5 phr few layer graphene in NR. With 1.5 phr sample, an increment of 19% in modulus was also observed. Further increase in loading of few layer graphene resulted in deterioration of properties. Interestingly, the % elongation at break remains unchanged. These results confirm that the observed improvement in the tensile properties of few layer graphene-NR nanocomposites is a consequence of the unique structure and property of this material. The property improvements are similar to what was earlier observed with few layer graphene prepared using melamine as the exfoliating agent^68^. The curcumin present in the nanocomposite could be extracted out of the cured nanocomposite thin film. The recovered curcumin had a chemical structure identical to the original sample used for exfoliation implying that the exfoliating agent did not undergo any chemical transformation during the process of preparing few layer graphene or the nano-composite (SI Section S8.2).

**Table 3:** Tensile properties of cured few layer graphene NR thin film nanocomposites.

Category		Sample	Tensile strength (MPa)	Tensile Modulus (MPa)	Elongation at break (%)
Controls		NR Latex	25.0 ± 1.5	1.6 ± 0.03	869
		Curcumin Control	25.3 ± 1.2	1.4 ± 0.03	875
		Control (0.7 phr Graphite)	25.9 ± 2.0	1.5 ± 0.02	856
		1.5 phr carbon black (HAF 330)	26.1 ± 1.6	1.4 ± 0.02	861
		1.5 phr carbon black (SAF 220)	27.4 ± 1.7	1.5 ± 0.02	860
		1.5 phr carbon black (SRF)	25.2 ± 1.5	1.4 ± 0.02	854
Few layer graphene (Graphite:Curcumin:Darvan)-NR thin film composites		0.3 phr Graphene	29.0 ± 1.7	1.6 ± 0.03	860
		0.7 phr Graphene	30.8 ± 1.8	1.6 ± 0.03	854
		1.5 phr Graphene	33.9 ± 2.0	1.9 ± 0.03	841
		3 phr Graphene	26.4 ± 1.5	1.7 ± 0.03	864
		5 phr Graphene	25.9 ± 1.5	1.7 ± 0.03	863
Reported values of GO or rGO/NR nanocomposites	NR/GO composites using ULMR process^60^.	Control	17.1	2.4	579
		2 wt% Graphene	25.2	6.6	564
	NR/exfoliated GO nanocomposite^62^.	Control	20.6	1.4	745
		5 wt% GO	27.9	5.0	657
	NR/exfoliated GO nanoplatelets composites^66^.	Control	2.34	1.6	—
		1 wt% GO	3.13	2.3	—
	Graphene oxide/NR latex based elastomer composites^71^.	Control	9.35	1.5	1088
		0.08 wt% GO	12.68	1.6	1169
Reported values of few or multi layers graphene/NR nanocomposites	Defect free few layer (2–5) graphene-NR latex^68^.	Control	25.0	1.6	869
		1.43 wt% of defect free few layer graphene	34.9	2.0	820
	Multi layers graphene/NR nanocomposites^55^.	Control	5.73	0.96	635
		3 wt% of multi-layers graphene	12.5	5.01	498

**Figure 7:**
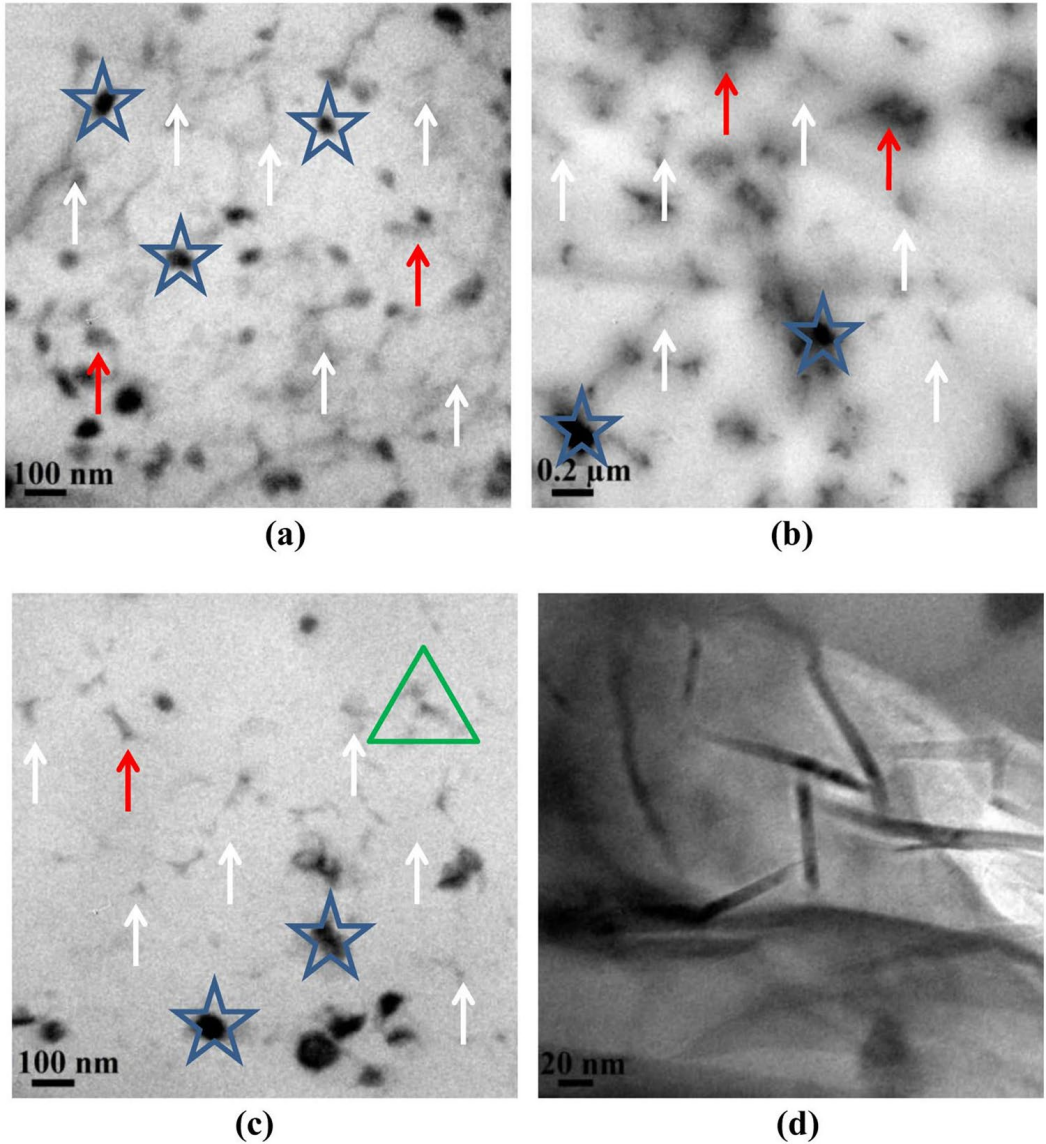
(**a**–**c**) are the TEM images of graphene reinforced NR latex thin films and (**d**) HR-TEM image of the portion marked as green triangle in Fig. 7c.

The TEM images of 1.5 phr few layer graphene-NR thin film nanocomposite are shown in Fig. 7a–d. The images display network like structures and the graphene appears mostly in an exfoliated form. Uniform and homogeneous distribution of few layers of graphene (white arrows) in the NR latex matrix is also apparent. Stacked graphene (>10 layer, red arrows) and agglomerated graphene (blue stars) are also evident in the TEM images. The later is due the strong forces of attraction between individual graphene layers/sheets^64^. The HR-TEM image (Fig. 7d) of the area in green triangle discloses the existence of exfoliated graphene in the NR matrix with a thickness of approximately 5–10 nm.

**Figure 8:**
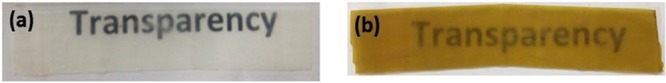
Relative transparency of (**a**) NR latex (control) thin film and (**b**) graphene NR latex nanocomposites thin films (1.5 phr).

The nano-composite containing 1.5 phr few layer graphene is transparent (Fig. 8b) and possesses a transparency comparable to NR latex thin film (Fig. 8a). However, addition of few layer graphene resulted in yellowing of the sample, most likely due to the presence of curcumin.

### Biocompatibility studies

The few layer graphene-NR latex thin film was examined for its biocompatibility properties. An *in-vitro* cytotoxicity study was performed on the thin film nanocomposite (1.5 phr graphene incorporated NR latex thin film) extracts using Balb/c3T3 cell lines. We observed that the cells treated with the negative control did not induce any cytotoxicity while the positive controls persuaded severe cytotoxicity (Table 4). The investigation also show that the undiluted and 1:2 diluted 1.5 phr graphene incorporated NR latex thin film nanocomposite show toxicity. However, in other dilutions (1:4, 1:8, 1:16 and 1:32), no cytotoxicity was observed. This underpinning degree of cytotoxicity is, however, acceptable in terms of biological safety evaluation of NR latex thin film based products such as hand gloves and condom.

**Table 4:** Cytotoxicity results obtained for 1.5 phr graphene incorporated NR latex thin film.

Dilution	Confluent monolayer (+ is present and − is absent)	toxicity	Grade
(Untreated 1 × DMEM medium)	+	None	0
Undiluted	−	Severe	4
1:2	−	Severe	4
1:4	+	None	0
1:8	+	None	0
1:16	+	None	0
1:32	+	None	0
(Thin films from natural Rubber latex gloves)	−	Severe	4

Grade 0 refers the toxicity is none and indicates discrete intracytoplasmatic granules, no cell lysis and no reduction of cell growth.

Grade 4 mentions the toxicity is severe and show the nearly complete or complete destruction of the cell layers.

Skin irritation is a key toxicity endpoint to assess biocompatibility of medical devices. Therefore, an *in-vivo* skin irritation was performed to the thin film nanocomposite using New Zealand white Rabbits. The experiments show that no mortality and morbidity was observed in any of the animals used. In addition, no significant change in body weight was observed at the end of the experiment (Table 5).

**Table 5:** Individual body weights and body weight changes of the New Zealand white Rabbits.

Rabbits number	Individual body weights (g)		Increase in body weight (g)
	At the start of experiments	At the end of experiments	
1	2708.9	2712.5	3.6
2	2692.0	2695.8	3.8
3	2699.0	2703.0	4.0

Individual score for erythema/eschar and oedema of the test site and control site after 1, 24, 48 and 72 h was also calculated (Table 6). All erythema grades plus oedema grades (24 ± 2) h, (48 ± 2) h, (72 ± 2) h was added separately for nanocomposite thin film and control for each animal. The calculated grades are appeared as zero, which indicates that the thin film nanocomposite did not cause any skin irritation to the Rabbits.

**Table 6:** Calculated grades of skin irritation of the thin film nanocomposite (T) and negative control (C).

Skin Reaction	Observation Time (h)	Individual score					
		Rabbit No. 1		Rabbit No. 2		Rabbit No. 3	
		C	T	C	T	C	T
Erythema and Eschar formation	1	0	0	0	0	0	0
	24	0	0	0	0	0	0
	48	0	0	0	0	0	0
	72	0	0	0	0	0	0
Oedema formation	1	0	0	0	0	0	0
	24	0	0	0	0	0	0
	48	0	0	0	0	0	0
	72	0	0	0	0	0	0

Sensitization (Type IV allergy) is a main toxicity endpoint to assess biocompatibility of medical devices and Guinea pig maximization test is the preferred method to determine the sensitization potential of a given medical devices. Therefore, an *in-vivo* skin sensitization potential of graphene reinforced NR latex thin films was evaluated using the Guinea Pig Maximization test. Skin reaction grading was performed at 24 and 48 h after removing the challenge patch using a Magnusson and Kligman scale (Table 7). A comparison of the skin reactions elicited in terms of incidence and severity were made to determine whether the nanocomposite thin film induces sensitization. The susceptibility of these strains of the Guinea pigs to a proven sensitizing agent i.e. α-Hexylcinnamaldehyde has also been established (Table S8). The experiments show that there were no statistically significant mean weight differences in bodyweights between the control and the treated groups from the first day to the end of the experiment (Table 8). The observed results suggested that the Guinea Pig treated with the thin film nanocomposite extracts did not show any sensitization reactions. Thus, these biological evaluations suggest that this graphene incorporated NR latex nanocomposite thin film could be used to produce commercially important health care products.

**Table 7:** Results of grading of skin reaction (sensitization) after removal of the challenge patch.

Group	Animal No.	Magnusson Kligman Scale			
		24 h		48 h	
		Challenge phase	Tophical induction phase	Challenge phase	Tophical induction phase
G1	1	0	0	0	0
	2	0	0	0	0
	3	0	0	0	0
	4	0	0	0	0
	5	0	0	0	0
G2	6	0	0	0	0
	7	0	0	0	0
	8	0	0	0	0
	9	0	0	0	0
	10	0	0	0	0
	11	0	0	0	0
	12	0	0	0	0
	13	0	0	0	0
	14	0	0	0	0
	15	0	0	0	0
G3	16	0	0	0	0
	17	0	0	0	0
	18	0	0	0	0
	19	0	0	0	0
	20	0	0	0	0
G4	21	0	0	0	0
	22	0	0	0	0
	23	0	0	0	0
	24	0	0	0	0
	25	0	0	0	0
	26	0	0	0	0
	27	0	0	0	0
	28	0	0	0	0
	29	0	0	0	0
	30	0	0	0	0

**Table 8:** Body weights of the animals used for skin sensitization.

Group No.	No. of Animals	Weight (g)		
		At the start of experiment	At the end of experiment	Increase in weight
1	5	423.3 ± 14.3	461.8 ± 14.0	38.5 ± 0.3
2	10	416.5 ± 21.3	455.3 ± 21.1	38.7 ± 0.3
3	5	451.6 ± 16.2	489.7 ± 15.8	38.1 ± 0.4
4	10	407.0 ± 27.5	445.7 ± 27.1	38.7 ± 0.4

## Conclusions

Naturally available molecules, such as, curcumin and tetrahydrocurcumin are found to be an excellent exfoliating agents for graphite and produces defect free few layers graphene. An efficient exfoliation shall be achieved in both solid and liquid phase. A widely available Sand grinder can be used for purposes of exfoliation making such processes robust and easily scalable. Using computational methods, it is proposed that non covalent interaction of curcumin with graphene contributes to the stabilization of the layers of graphene. Aqueous dispersions of curcumin exfoliated few layer graphene (produced by solid-phase exfoliation) was used to prepare NR thin film nano-composites. The graphene-NR nanocomposites exhibit a 36% increase in tensile strength at 1.5 phr loading of graphene. Biocompatibility studies viz. *in-vitro* cellular toxicity and *in-vivo* skin sensitization and irritation show that the produced graphene–natural rubber thin film nanocomposite are safe from a cytotocxictiy and skin irritation point of view. The simplicity of the method, the general safety of the exfoliating agents employed, the useful properties obtained in thin film nanocomposites and its biocompatibility, make this approach an interesting and useful method to produce commercial products, which come in contact with body parts or body fluids.

## Experimental

### Exfoliation of graphite with curcumin, tetrahydrocurcumin and quercetin

Solid-phase exfoliation of graphite with Curcumin:Tetrahydrocurcumin:Quercetin was performed in a planetary ball mill (A Retsch PM 400 with 4 grinding bowl fasteners). The grinding was carried out in Ytrria stabilized zirconia jars with zirconia balls. A typical procedure consisted of grinding (i) curcumin or (ii) tetrahydrocurcumin (both, purchased from Somu Chemicals, India) or (iii) quercetin (purchased from Otto Chemie Pvt. Ltd, India) with graphite (purchased from Aldrich) (at a weight ratio of 3:1) at 100 rpm for 1 h (successive grinding for 1 h with 15 min. grinding and 15 min. pause). Darvan (Sodium polynaphthalene sulphonate) was added in 12.5 wt% (with respect to graphite) during the grinding process. The exfoliated graphite thus obtained (Graphite:Curcumin:Darvan or Graphite:TetrahydroCurcumin:Darvan or Graphite:Quercetin:Darvan) was made into a 30 wt% solution in de-ionized water using probe sonication technique (750 W for 2 min at 25% amplitude).

Liquid-phase exfoliation of graphite with curcumin in acetone and water mixture was also performed in a similar fashion. A, 30 wt% of Graphite:Curcumin:Darvan (1:3:0.125) in varying proportions of acetone de-ionized water mixtures was used as the liquid phase.

Sand grinding of 1:3:0.125 mixture of Graphite:Curcumin:Darvan was performed in Diamill S0.3 supplied by Abigail Enterprises, India and having a grinding chamber volume of 100 mL. The outlet of the grinder was connected to a refrigerated chiller. In this case, Graphite:Curcumin:Darvan mixture was made into a 30 wt% dispersion in de-ionized water and fed into the sand grinding mill and ground for 1 h. 100 g of zirconia balls having 0.85 mm diameter was used.

### Preparation of few layer graphene-NR thin film nano-composites

Solid-phase exfoliated graphene with curcumin (Graphite:Curcumin:Darvan; 1:3:0.125) was prepared as an aqueous dispersion (30 wt%) and added to [0.3, 0.7, 1.5, 3 and 5 phr (parts/100 g of rubber) concentrations] compounded NR latex. Probe sonication (750 W/3 min/20% amplitude) was used for ensuring uniform mixing of the filler into the NR latex. A simple two step dipping procedure using a lab model dipping machine was employed to produce nanocomposites. The samples were cured in hot air oven for 45 min at 80 °C. Silica powder was used to strip out the dipped samples from the glass moulds (detailed dipping procedure is explained in the supporting information). The cured samples were then allowed to mature for 2–3 days at room temperature. Ring samples were cut for tensile property measurement according to ASTM-D412.

### Characterization of samples

XRD was carried out on XEUSS SAXS/WAXS system using a Genix micro source from Xenocs operated at 50 kV and 0.6 mA. The Cu Kα radiation (wavelength = 1.54 Å) was collimated with FOX2D mirror and two pairs of scatter less slits from Xenocs. The 2D-patterns were recorded on a Mar345 image plate and processed using Fit2D software. All the measurements were made in the transmission mode.

Horiba Scientific LabRAM-HR Raman microscope with an excitation laser of 514 nm and 1800 g/mm grating was used and the spectra were recorded with a 100× lens. Aqueous dispersions of Graphite:Curcumin:Darvan or Graphite:TetrahydroCurcumin:Darvan or Graphite:Quercetin:Darvan was drop cast onto a glass plate and allowed to dry at 70 °C. The glass plate was carefully dipped either in acetone (for Graphite:Curcumin:Darvan and Graphite:TetrahydroCurcumin:Darvan) or in methanol (for Graphite:Quercetin:Darvan) for 5 times to remove the exfoliating agents and dried at 70 °C for 1 h.

Graphene dispersions (Graphite:Curcumin:Darvan or Graphite:TetrahydroCurcumin:Darvan or Graphite:Quercetin:Darvan) were drop cast onto standard TEM grids for preparing samples for Transmission electron microscopy (TEM). A JEOL JEM-2010 was used to analyze the samples at 200 kV. In the case of nanocomposite samples, cryo-microtoming at −70 °C was employed to prepare the samples. Tensile testing of the ring samples were performed using a Shimadzu AGX-10 universal testing machine (UTM) at a cross head speed of 500 mm/min and load cell 500 N according to ASTM D412. 20–25 samples from each set (thickness ∼ 40–60 µm) were tested.

### Biocompatibility investigations

*In-vitro* cellular toxicity and *in-vivo* skin irritation and skin sensitization have been completed as per the standards of ISO 10993-1 biological evaluation and biocompatibility testing of medical devices.

An *in-vitro* cytotoxicity study was performed using Balb/c3T3 cell lines. The extract of the thin film nanocomposite (1.5 phr graphene incorporated NR latex thin film) was prepared using serum supplemented 1 × Dulbecco’s Modified Eagle’s (DMEM) cell culture medium at 37 °C for 24 h at the ratio of 6 cm^2^ of the composite thin film per mL of the medium. Thin film derived from NR latex gloves was employed as a positive control whereas the cell culture medium was used as a negative control. Balb/c3T3 cells were seeded in 96-well plate at approximately 1 × 10^4^ cells per well. On the day of treatment, the culture medium was removed and replaced with various dilutions of the nanocomposite thin film extracts (undiluted, 1:2, 1:4, 1:8, 1:16 and 1:32), negative control and positive controls. The cell cultures were then incubated at 37 °C for 24 h in an atmosphere of 5% CO_2_. Then, the cells were subjected to qualitative measurements viz., cell confluency and morphology; and grades (Tables S5 and 4) of cytotoxicity were assessed.

Healthy, adult New Zealand rabbits (weighting 2.6–2.7 kg, male) and healthy adult guinea pigs (weighting 360–470 g, female), were obtained from Sainath Agencies, Hyderabad, India. They were placed in stainless steel (rabbits) and polypropylene (guinea pigs) cages, provided with standard laboratory diet and water ad libitum. The animal facility was maintained at 18.7–22.6 °C, a relative humidity of 37–60%, and a 12 h light/dark cycle throughout the experiment. This study was approved by the **I**nstitutional **A**nimal **E**thics **C**ommittee [IAEC no. for the Skin Sensitization Test (IAEC-10th Jul 2014-Proposal 4) and Skin Irritation Test (IAEC-10th Jul 2014-Proposal 4)]. These studies were executed based on OECD Principles of Good Laboratory Practice.

An *in-vivo* skin irritation was performed to the thin film nanocomposite using New Zealand white Rabbits (3 Nos.). All the three rabbits were clipped free of hair on dorsal side from an area of approximately 10×15 cm on both sides of the spinal cord about approximately 18 h prior to commencement of the experiment. Size ∼6.25 cm^2^ thin film nanocomposite (in the dorsal region on the left cranial end and right caudal end) along with a positive control (absorbent gauze at the right cranial end and left caudal end) was applied topically to the three male Rabbits. The Rabbits were observed and evaluated for 3 consecutive days for morbidity & mortality, body weight, abnormal clinical signs and symptoms (Tables S6 and 5).

An *in-vivo* skin senzitization was completed to the thin film nanocomposite using guinea pigs (40 Nos). Polar (physiological saline) and non-polar (sunflower oil) extracts were prepared by extracting 6 cm^2^ of thin film nanocomposite per ml of solvent at 37 °C for 72 h. Animals were separated as four groups; (i) Physiological saline extract (10 Nos) (ii) Physiological saline control (5 Nos) (iii) Sunflower oil extract (10 Nos) and (iv) Sunflower oil control (5 Nos) (Table S7 and Figure S17). The susceptibility of these strains of guinea pigs to known sensitizing agent, α-Hexylcinnamaldehyde (Sigma Aldrich) has also been established as a positive control (Table S8). Induction of skin sensitization was a two-stage procedure with intradermal injections initially administered, followed by a closed topical patch exposure on day 7. Intradermal injections of the nanocomposite thin film extracts, vehicles and Freund’s Complete Adjuvant (FCA) in various mixtures were administered to the vehicle control and test groups. On day 6, following the intradermal injections, test area was treated with 0.5 mL of 10% sodium lauryl sulphate (Loba Chemie Pvt Ltd., Mumbai, India). On the next day, topical patch of size 8 cm^2^ (Ramaraju Surgical Cotton Mills Ltd., India) loaded with 0.5 mL of test item extract and vehicle, respectively was applied topically to respective groups of guinea pigs, on the same site as that of intradermal injections. This occlusive dressing was held in place for 48 h. Two weeks following the topical patch induction, challenge exposure was administered as a topical patch of size 8 cm^2^. Patch soaked with 0.5 mL of test item extract was applied on left side whereas the patch with 0.5 mL of vehicle was applied on right side of each animal in respective groups for 24 h at sites other than those used for intradermal injections/topical applications and the application sites were marked with non-irritant marker pen.

## Electronic supplementary material

Supporting Information
