# Supplementary material for: Facile, environmentally benign and scalable approach to produce pristine few layers graphene suitable for preparing biocompatible polymer nanocomposites
Source: Sci Rep. 2018 Jul 25;8:11228. doi: 10.1038/s41598-018-28560-1 (PMC6060110; doi:10.1038/s41598-018-28560-1)
Supplement: Supplementary file 1 — Supporting Information [file 41598_2018_28560_MOESM1_ESM.pdf]

Supporting Information

Facile, environmentally benign and scalable approach to produce pristine few layers graphene suitable for preparing biocompatible polymer nanocomposites

Gejo George[1], *Suja Bhargavan Sisupal*[1], *Teenu Tomy*[1], *Alaganandam Kumaran*[1], *Prabha Vadivelu*[2], *Vemparthan Suvekbala*[1], *Swaminathan Sivaram*[3] *and Lakshminarayanan Ragupathy*[1*]

[1]Corporate R&D Center, HLL Lifecare Limited, Akkulam, Sreekariam (P.O), Trivandrum 695017, India

[2]CSIR-National Institute for Interdisciplinary Science and Technology, Industrial Estate (P.O), Pappanamcode, Trivandrum 695019, India

[3]Polymers and Advanced Materials Laboratory, National Chemical Laboratory, Dr. Homi Bhabha Road, Pune 411008, India

[*]Corresponding author:laks@lifecarehll.com, laks77@gmail.com

Contents

S1 Planetary ball milling (dry grinding) of graphite with curcumin/tetrahydrocurcumin/quercetin as exfoliating agents

S1.1 Planetary ball milling technique

S2 Characterization of exfoliated graphite (using curcumin/tetrahydrocurcumin/quercetin as exfoliating agent) produced using planetary ball milling (solid phase exfoliation)

S2.1XRD

S2.2 Raman spectroscopy

S2.3 TEM analysis

S3 Characterization of exfoliated graphite (curcumin as exfoliating agent) produced using planetary ball milling (solution-phase exfoliation)

S3.1XRD

S3.2 Raman Spectroscopy

S3.3 TEM analysis

S4 Characterization of exfoliated graphite (curcumin as exfoliating agent) produced using sand grinder (solution-phase exfoliation)

S4.1XRD

S4.2 Raman Spectroscopy

S4.3 TEM analysis

S5 Toxicity of graphene exfoliating agents/chemicals/salts/etc.

S6 Computational studies on interaction of graphene with curcumin

S7 Preparation of few layer graphene-NR thin film nanocomposite

S8 Characterization of few layer graphene-NR thin film nanocomposite

S8.1TEM analysis

S8.2 Stability of curcumin under the processing condition

S9 Biocompatibility of graphene-NR latex thin film nanocomposite

S1 Planetary ball milling of graphite with curcumin/tetrahydrocurcumin/quercetin

The planetary ball mill used was a Restch PM400 with 4 grinding bowl fasteners. In a typical procedure 20 g of graphite was milled with 60 g curcumin/tetrahydrocurcumin/quercetin (1:3 ratio) and 2.5 g Darvan I (stabilizer) for 1 h at 100 rpm (15 min. grinding followed by 15 min. pause to avoid excessive heat production). Zirconia containers and zirconia balls were used for the ball milling procedure. After grinding, the samples are dispersed in water and sonicated for 2 min. at 25 % amplitude using a 750 W sonicator.

S2 Characterization of exfoliated graphite (curcumin/tetrahydrocurcumin/quercetin as exfoliating agent) produced using planetary ball milling (solid phase exfoliation)

S2.1 XRD of quercetin exfoliated graphite

The XRD of Graphite:Quercetin:Darvan (1:3:0.125 before and after grinding) sample and graphite is shown as Figure S1.

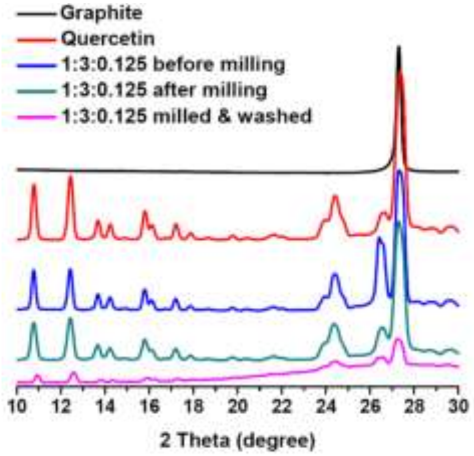

Figure S1: Normalized XRD of graphite and graphene produced using quercetin as the exfoliating agent (ratios correspond to Graphite:Quercetin:Darvan).

S2.2 Raman spectra of curcumin/tetrahydrocurcumin/quercetin exfoliated graphite

The Raman spectra of Graphite:Quercetin:Darvan sample and graphite is shown as Figure S2. The shape of 2D band 1:3:0.125 Graphite:Quercetin:Darvan shows that the produced graphenes are about 10 layers[1-3] thick.

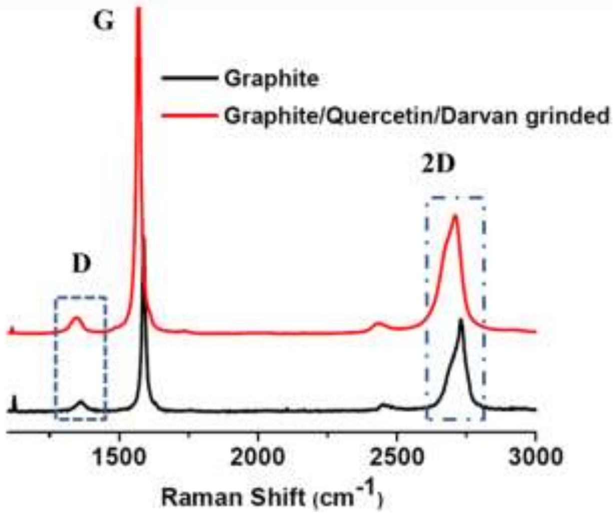

Figure S2: Raman spectra of graphene samples produced using quercetin as the exfoliating agent.

The 2D band of all Graphite:Curcumin:Darvan samples is shown as Figure S3. Here again, a clear shift of the 2D band to the lower wavelength is visible. The shape of 2D band in 1:3:0.125 Graphite:Curcumin:Darvan shows that the produced graphenes are few layers[1-5]. The zoomed in figures of D and 2D bands of 1:3:0.125 Graphite:Curcumin:Darvan are shown in Figure S4.

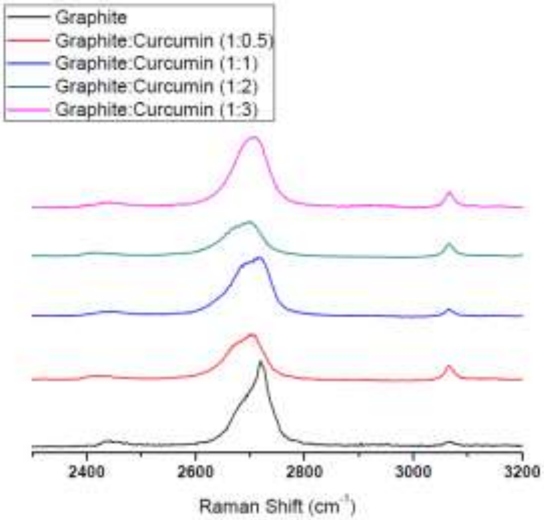

Figure S3: 2D band of graphite and graphite/curcumin sample milled at different ratios. The Darvan ratio is 0.125 in all the cases.

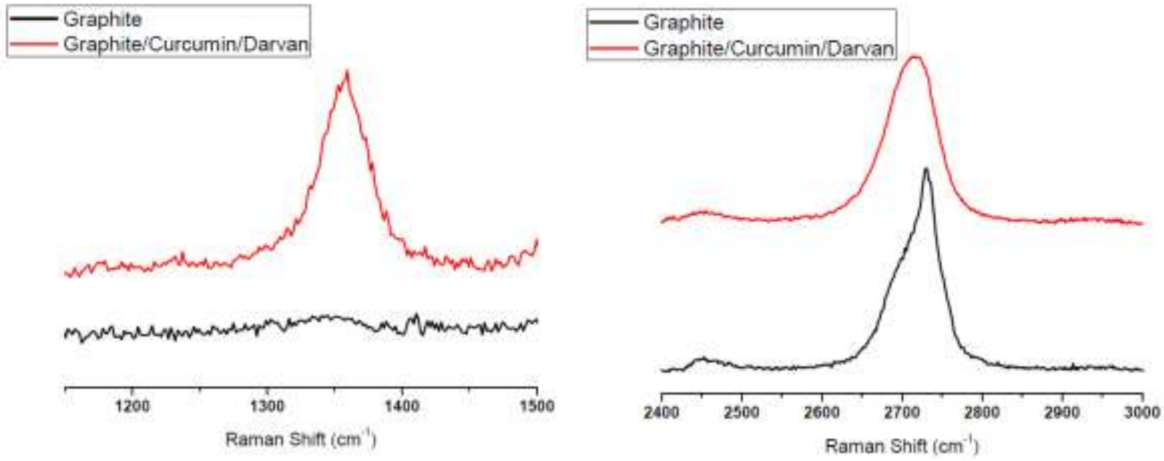

Figure S4: D and 2D bands of graphite and milled graphite/curcumin/Darvan (1:3:0.125) samples.

The 2D band of all Graphite:TetrahydroCurcumin:Darvan samples is shown as Figure S5. Here again, a clear shift of the 2D band to the lower wavelength is visible. The shape of 2D band

1:3:0.125 Graphite:TetrahydroCurcumin:Darvan shows that the produced graphenes are few layer[1-3].

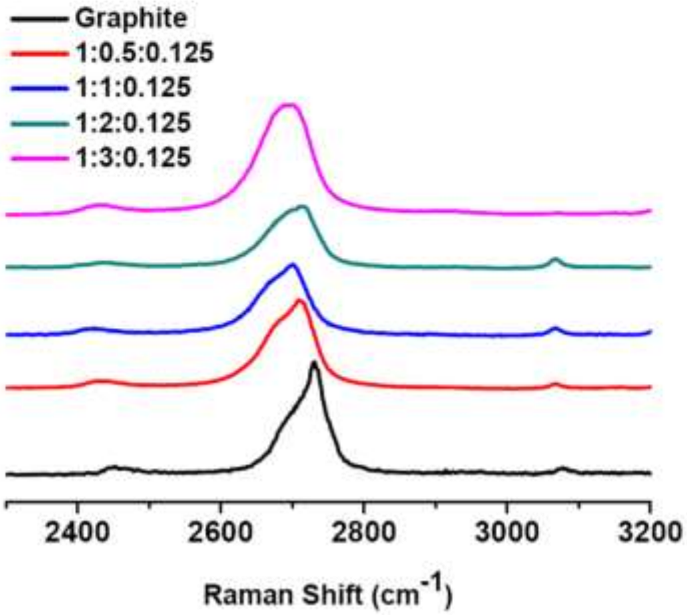

Figure S5: 2D band of graphite and graphite/tetrahydrocurcumin/darvan sample milled at different ratios. The ratios correspond to Graphite:tetrahydrocurcumin:darvan.

The deconvolution of the 2D band in 1:3:0.125 Graphite:Curcumin:Darvan sample leads to four lorenztian peaks at 2666.31, 2688.46, 2708.88 and 2725.91 cm^{-1} and the deconvolution of 2D band of the 1:3:0.125 Graphite:TetrahydroCurcumin:Darvan yields four lorenztian peaks appearing at 2664.87, 2684.26, 2701.85 and 2721.23 cm^{-1}. I_D/I_D', I_D/I_G and in-plane crystallite sizes, La of graphene samples prepared are given in Table S1.

Table S1. $I_D/I_{D'}$, I_D/I_G and in-plane crystallite sizes, La of graphene samples prepared using different exfoliating agents.

S. No:	Sample details	I_D/I_D'	I_D/I_G	L_a (nm)
1.	Graphite:Curcumin:Darvan(1:3:0.125)	0.85, 1.23, 1.09	0.54, 0.45, 0.40	28, 37, 41
2.	Graphite:Tetrahydrocurcumin/Darvan (1:3:0.125)	0.97, 0.74, 0.81	0.28, 0.21, 0.35	59, 79, 47
3.	Graphite:Quercetin:Darvan(1:3:0.125)	No. of graphene layers is 10, therefore, D' peak is not observed here.	0.17, 0.10, 0.21	98, 167, 79

S2.3 TEM analysis

The TEM images of 1:3:0.125 Graphite:Quercetin:Darvan sample is shown as Figure S6. The images clearly show that the produced graphene is multilayered (≥ 5) evidenced by the reduced transparency of the graphene sheets.

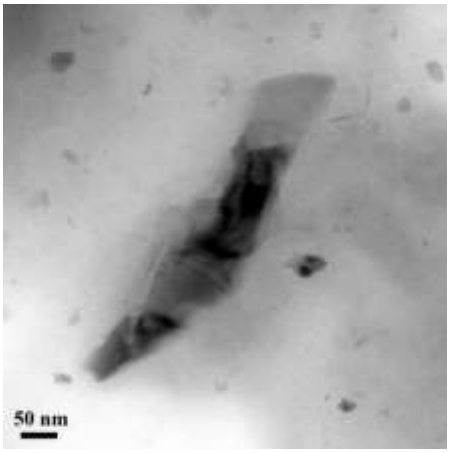

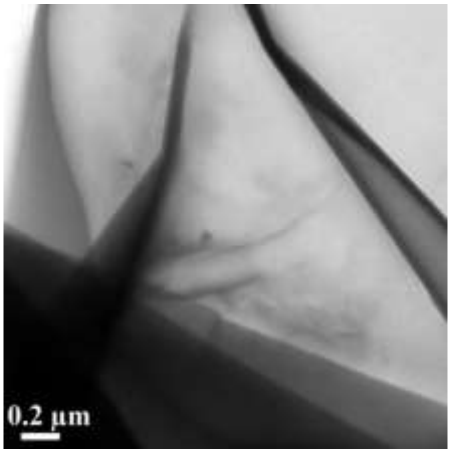

Figure S6: TEM images of 1:3:0.125 Graphite:Quercetin:Darvan sample.

S3 Characterization of exfoliated graphite produced using planetary ball milling (solution phase exfoliation)

S3.1 XRD

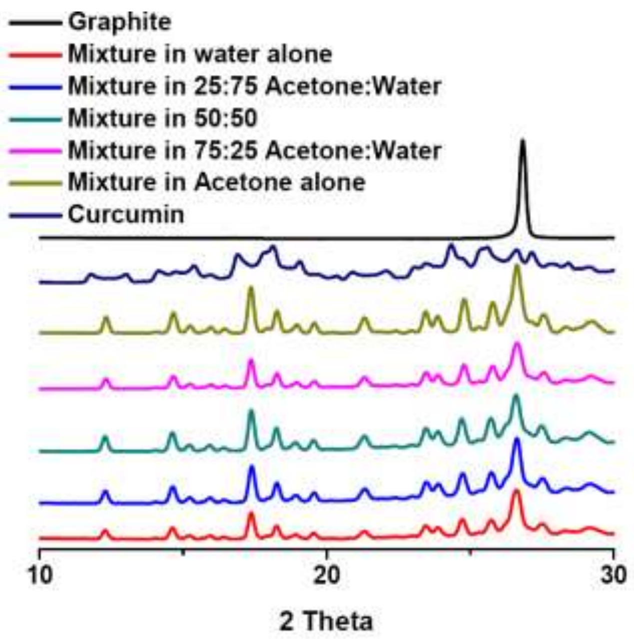

Figure S7: XRD of graphite, curcumin and Graphite:Curcumin:Darvan (mixture) samples with varying solvent ratios.

The normalized XRD spectrum of graphite, curcumin & Graphite:Curcumin:Darvan samples with varying solvent ratios (acetone:water) is shown as Figure S7.

S3.2 Raman Spectra

The Raman spectra of Graphite:Curcumin:Darvan samples prepared using different solvent ratios (acetone:water) are shown as Figure S8. This demonstrates that in the case of Graphite:Curcumin:Darvan with 50:50 (acetone: water) mixture the graphene produced is a bi-layer whereas in other solvent ratios it is found to be approximately 5 layers (2D band deconvolutes into 2 Lorentzian peaks)[1-3]. $I_D/I_{D'}$, I_D/I_G and in-plane crystallite sizes, La of graphene samples prepared using 50:50 (acetone: water) solvent mixture Graphite:Curcumin:Darvan is shown as Table S2.

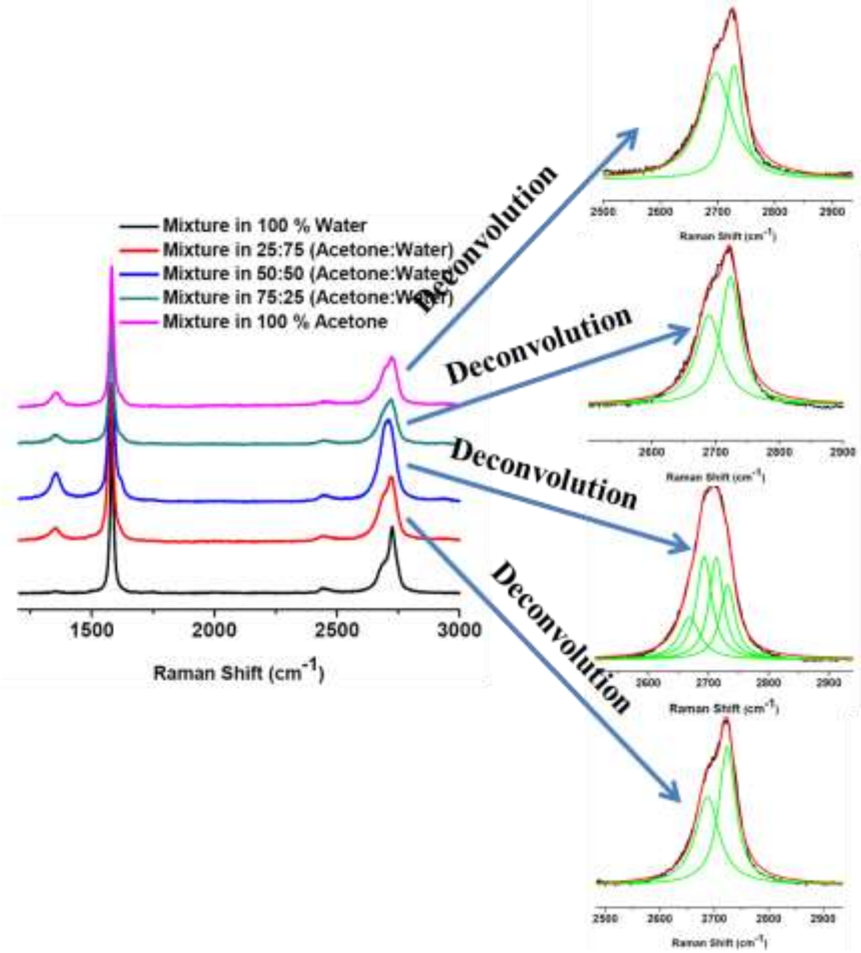

Figure S8: Raman spectra of Graphite:Curcumin:Darvan (mixture) samples with varying solvent ratios (acetone: water).

Table S2. $I_D/I_{D'}$, I_D/I_G and in-plane crystallite sizes, La of graphene samples prepared using wet grinding method in 50:50 wt% acetone:water using curcumin.

S. No.	Sample details	I_D/I_D'	I_D/I_G	L_a (nm)
1.	Graphite:Curcumin:Darvan (1:3:0.125)	0.88, 0.92, 1.12	0.16, 0.20, 0.23	104, 83, 73

$I_D/I_{D'}$ values clearly indicates that no new defects are introduced into the graphene samples during the grinding process.

S3.3 TEM analysis

TEM images of Graphite:Curcumin:Darvan (50:50 acetone:water) system with 1:3:0.125 ratio is shown as Figure S9.

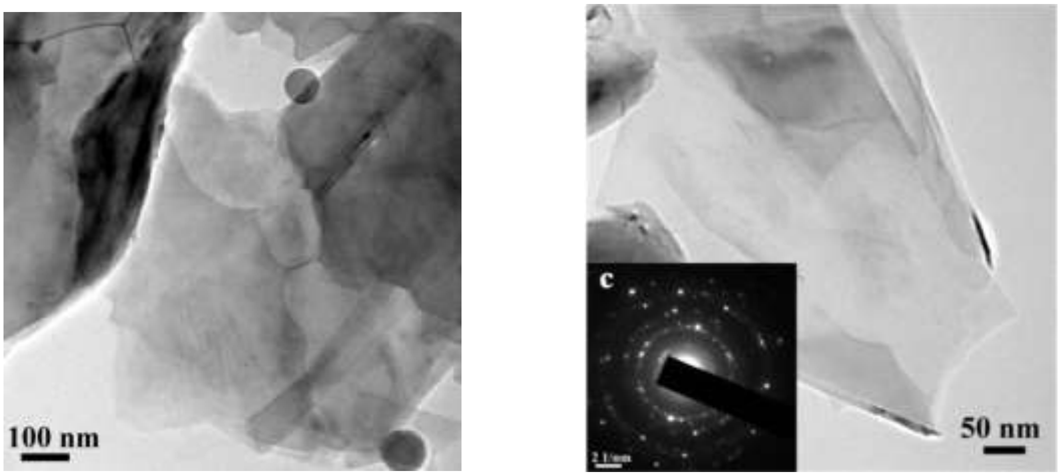

(a) (b)

Figure S9: TEM images of (a) & (b) Graphite:Curcumin:Darvan (50:50 actone: water) sample and c) SAED pattern

S4 Characterization of exfoliated graphite produced using sand grinder

S4.1 XRD

The normalized XRD of graphite and Graphite:Curcumin:Darvan (1:3:0.125) sample produced using sand grinding technique is shown as Figure S10.

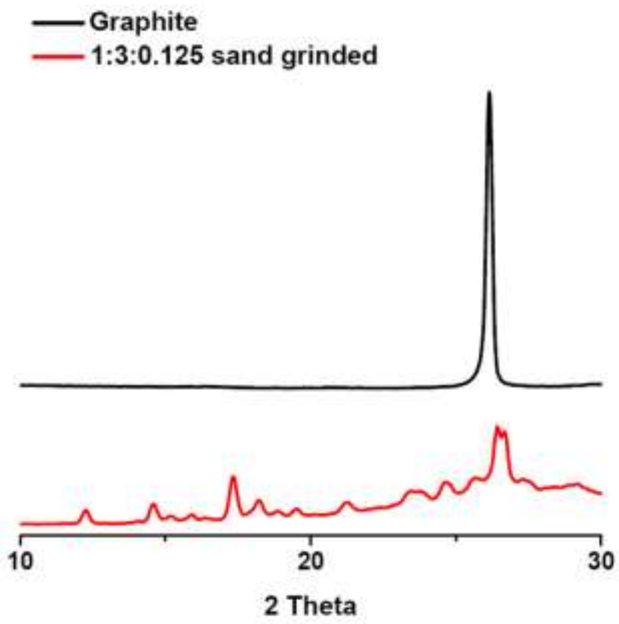

Figure S10: Normalized XRD of graphite and Graphite:Curcumin:Darvan (1:3:0.125 wt. ratio) sample produced using sand grinding technique.

S4.2 Raman Spectra

The Raman spectra of graphite and graphene (Graphite:Curcumin:Darvan,1:3:0.125) produced using sand grinding process, along with the deconvolution of 2D band is shown as Figure S11 and the corresponding I_D/I_G ratios in table S3.

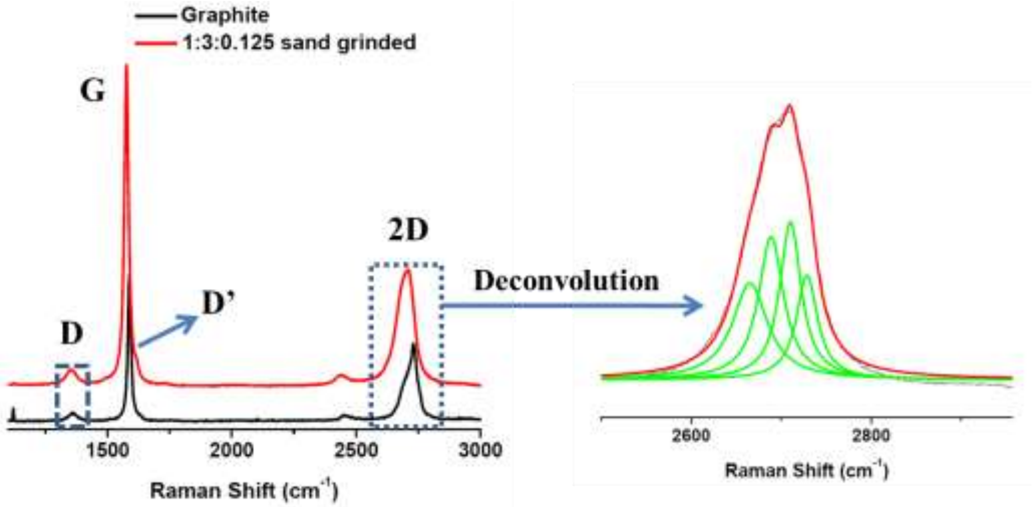

Figure S11: Raman spectra of graphite and Graphite:Curcumin:Darvan (1:3:0.125) sample produced using sand grinder and the deconvoluted 2D band.

Table S3. $I_D/I_{D'}$, I_D/I_G and in-plane crystallite sizes, La of graphene samples prepared using sand grinder.

S. No.	Sample details	I_D/I_D'	I_D/I_G	L_a (nm)
1.	Graphite:Curcumin:Darvan (1:3:0.125)	0.78, 0.55, 0.48	0.11, 0.10, 0.05	152, 167, 335

S.4.3 TEM analysis

The TEM images of Graphite:Curcumin:Darvan sample (1:3:0.125) produced by sand grinding technique is shown as Figure S12.

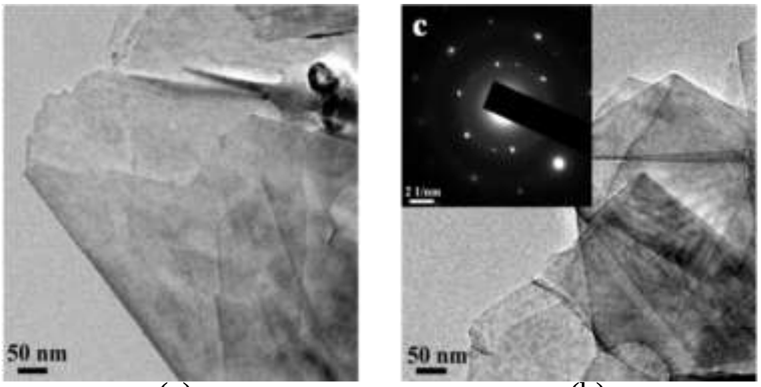

(a) (b)

Figure S12: (a) & (b) TEM images of Graphite:Curcumin:Darvan (1:3:0.125) samples produced by sand grinding and (c) SAED pattern of the graphene sheet.

S5. Table S4. Toxicity of graphene exfoliating agents/chemicals/salts etc.

S. No.	Exfoliation Method/agent	Reported no. of layers	Graphene production rate(yield) in g/hr	D/G Ratio	Toxicity		
					**Oral toxicity**	**Skin toxicity**	**Chronic toxicity**
1	**Solid-phase:** (a) Ball milling with Curcumin(Current work, solid-phase exfoliation, solution-phase exfoliation).<br>(b) sand Grinding with Curcumin	2-10	(a) 45.7 (solid-phase), 20 (solution-phase).<br>(b) 10 (sand grinding).	(a) 0.40-0.54 (solid-phase), 0.16-0.23 (solution-phase).<br>(b) 0.05-0.11 (sand grinding).	LD_{50}Oral-Mouse-<br>> 2,000 mg/kg.<br>LD_{50}Oral-Rat-<br>> 2,000 mg/kg.[54]	Slightly hazardous in case of skin contact (irritant).[55]	Curcumin, administered before or after radiation, markedly reduced acute and chronic skin toxicity in mice ($p < 0.05$).[56]
2	**Solid-phase:** Ball milling with Tetrahydrocurcumin(Current work)	2-10	45.7	0.21-0.35	LD_{50} Oral-Mouse- 300 mg/kg.<br>LD_{50}Oral-Rabbit-3200 mg/kg.<br>LD_{50} Oral-Rat-980 mg/kg.[57]		
3	**Solid-phase:** Ball milling with Quercetin and (Current work)	10	45.7	0.10-0.21	LD_{50}Oral-Rat-161 mg/kg.[58]		
4	**Solution-phase:** Shear exfoliation with N-methylpyrrolidone, aqueous solutions of sodium cholate and polyvinyl alcohol (PVA)[5].	Few layers	5.3	0.17-0.37	**N-methylpyrrolidone**<br>LD_{50}Oral-Rat-3,914 mg/kg.[59]<br><br>**Sodium cholate**<br>-<br>**PVA**<br>LD_{50}Oral-Rat-> 20,000 mg/kg.[60]	**N-methylpyrrolidone**<br>LD_{50} Dermal-Rabbit-8,000 mg/kg.[59]<br><br>-<br><br>-	

5	**Solid-phase:** Ball milling with Melamine [1,6].	Few layers	0.015[1] and 22.9[6]	0.41-0.49	LD_{50}Oral-Rat-male- 3,161 mg/kg.[61]	LD_{50}.Dermal - Rabbit - > 1,000 mg/kg.[11] No skin irritation.[61] Acute dermal toxicity in rabbits presents when the exposure is 1g/kg body wt.[62]	Long-term exposure to melamine reduces fertility and results in fetal toxicity in animal studies. The most commonly reported chronic renal toxicity is stone formation.[62]
6	**Solution-phase:** Wet stirred media milling with sodium dodecyl sulfate [7].	Single to Multilayers	1.5-2.5	~0.6-0.7 (532nm)	LD_{50} Oral-Rat-male and female- 1,200 mg/kg.[63]	Skin irritation (Rabbit 24 h OECD Test Guideline 404).[63] In animal studies SDS appears to cause skin and eye irritation.[64]	SDS may worsen skin problems in individuals with chronic skin hypersensitivity.[64]
7	**Solution-phase:** CVD with sodium ethoxide[8].	Few (4) layers	1	~1 (532 nm)	LD_{50} Oral-Rat-male and female - 598 mg/kg (OECD Test Guideline 401).[65]	Causes severe burns on skin (Rabbit -3 min OECD Test Guideline 404).[15] Create redness, pain blisters and skin burns.[66]	
8	**Solution-phase:** KI ntercalation with acid (HNO_3 and H_2SO_4)-intercalated/ exfoliated graphite[9].	30-40	0.9 g	No Raman	LD_{50} Intraperitoneal-Mouse- 700 mg/kg.[67]	Exposure to radiation can damage the basal cell layer of skin, resulting in inflammation, erythema, and dry or moist desquamation.[68]	
9	**Solution-phase:** Interlayer	1-3	0.4 g	No Raman	**$FeCl_2$** LD_{50} Oral-Rat-	**$FeCl_2$** No skin	**H_2O_2** Material is

	catalytic exfoliation with $FeCl_2$ and H_2O_2[10].				> 500 mg/kg.[69]<br><br>**H_2O_2**<br>Oral-Rat- 910 mg/kg.[70]	irritation (Rabbit-OECD Test Guideline 404).[69]<br>**H_2O_2**<br>LD_{50}Dermal-Rat- 4060 mg/kg<br>Very hazardous in case of skin contact(irritant).[70]	destructive to tissue of the mucous membranes and upper respiratory tract. Exposure can cause lung irritation, chest pain and oedema which may be fatal.[70]
10	**Solution-phase:** Na in liquid NH_3 Reduction[11].	1	0.33 g	~ 1.4 (514 nm)	**Ammonia**<br>LC_{50} Inhalation - Rat -4h - 2000 ppm.[71]<br>LC_{50} Rabbit inhalation 7,050 mg/cu m/1 hr.[72]<br>LD_{50} Rat oral 350 mg/kg.[73]<br>LD_{50}Mouse inhalation 4,837 ppm/1 hr.[74]	**Ammonia**<br>The vapor even in low concentration is extremely irritating to skin, eyes and respiratory passages.[75]	**Ammonia**<br>Exposures of 500 ppm for 30 min have caused upper respiratory irritation, tearing, increased pulse rate, and blood pressure.[76]
11	**Solution-phase:** Exfoliation with chlorosulfonic acid and H_2O_2[12].	Few layers (3)	~0.25 g	0 (514 nm)	**chlorosulfonic acid**<br>LC_{50} mouse inhalation 52.5 mg/cu m/2 hr.<br>LC_{50} rat inhalation 38.5 mg/cu m/4 hr.[77]	**chlorosulfonic acid**<br>Highly irritating & corrosive to eyes, skin, mucous membranes.[78]	
12	**Solution-phase:** Sonication of expanded graphite with 1-pyrenesulfonic acid sodium salt (Most effective one)[13].	Few layer	0.02	~0.33	-	-	-

13	**Solution-phase:** Sonication with Isopropanol.[14]	1-5	~ 0.015	0.2-0.4 (633 nm)	LD_{50} Oral-Rat- 5,045 mg/kg Remarks: Behavioral: Altered sleep time (including change in righting reflex). Somnolene (general depressed activity). LC_{50} Inhalation-Rat- 8 h - 16000 ppm.[79]	Mild skin irritant. LD_{50} Dermal - Rabbit - 12,800 mg/kg.[79]	Repeated exposures produced toxic effects only at the highest concentration (5000 ppm) and a kidney change in male rats of unknown biological significance.[80]
14	**Solution-phase:** Horn ultrasonication of naturally occurring graphite flakes with bile salt sodium cholate[15].	Few layers	0.018	-	-	-	-
15	**Solution-phase:** Sonication with *N*-Methyl-2-pyrrolidone (NMP) and thermal treatment[16].	1-4	<0.01	< 0.15 (514 nm)	LD_{50} Oral-Rat- 3,914 mg/kg LDLo Inhalation -Rat - 4 h - > 5100 ppm.[81]	LD_{50}Dermal - Rabbit - 8,000 mg/kg.[81]	The no-observed-effect level for NMP was 5000 ppm for male and female rats, 600 ppm for male mice, and 1200 ppm for female mice.[82]
16	**Solution-phase:** Intercalation of alkali metal (ternary KCl-NaCl-$ZnCl_2$eutectic system) between graphite Interlayers[17].	Few layers (18% of are single/double layer)	0.012	0.15 (514 nm)	-	-	-
17	**Solution-phase:** Sonication with Gum Arabic (biopolymer)[18].	5-20	$\sim 6\times10^{-3}$	~0.25 (633 nm),	LD_{50} Oral-Rat- > 16,000 mg/kg.[83]	-	-

18	**Solution-phase:** Grinding with ionic liquids (1-Butyl-3-methylimidazoliumhexafluorophosphate)[19.]	2-5	0.01	0.23 (514 nm)	-	-	-
19	**Solution-phase:** Dissolution in superacids (chlorosulphonic acid)[20a&20b].	Single (70%)	~4.2×10^{-3}	0.1-0.5 (514 nm)	LC_{50} mouse inhalation 52.5 mg/cu m/2 hr.[84]	Highly irritating & corrosive to eyes, skin, mucous membranes.[85]	-
20	**Solution-phase:** Sonication inCyclohexanone[21].	Few	3 $x10^{-3}$	No Raman	LD_{50} Oral-Rat-12,705 mg/kg LC_{50} Inhalation -Rat-4 h-34,000 mg/l (OECD Test Guideline 403) LD_{50} Dermal-Rabbit - > 2,000 mg/kg.[86]	No skin irritation.[86]	Chronic or repeated exposure can result in skin irritation due to defatting of the skin.[87]
21	**Solution-phase:** Sonicationwith Sodium dodecylbenzenesulfonate.[22]	Few layers (>40% and Mono layer (~3%)	$2.5x10^{-3}$	0 on thick films (large FLG), ~ 0.4 on thin films (small flakes), 532 nm	LD_{50} Oral-Rat-438 mg/kg.[88]	Skin irritatant.[88]	-
22	**Solution-phase:** Sonication with vinylcaprolactam.[23]	Few layers (5-7)	$2.1x10^{-3}$	~0.l4 (532 nm), 0.1 in powder	LD_{50} Oral- Rat-male/female1,860 mg/kg.[89]	LD_{50} Dermal-Rabbit-male and female - 1,700 mg/kg (OECD Test Guideline 402).[90]	Repeated exposure tosmallquantitiesmayaffectcertainorgans.Damagestheliver.[89]

23	**Solution-phase:** Grinding with Ionic liquid (1-hexyl-3-methylimidazolium hexafluorophosphate) followed by sonication[24].	Few layers	2.2 x 10^{-3}	~0.l4 (532 nm), 0.1 in powder	-	Causes skin irritation.[91]	-
24	**Solution-phase:** Sonication with sodium chloate surfactant[25].	Bi and Multi layers	2 x10^{-3}	0 (488 nm)	LD_{50}Rat-1200 mg/kg.[92]	Hazardous in case of skin contact (irritant).[92]	-
25	**Solution-phase:** High-shear mixing with orthodichloro benzene and(DCB) sonication[26].	Few layers (<5)	2.0x10^{-3}	<0.3 (514 nm)	LD_{50} Rat-oral 1516-2138 mg/kg.[93] LD_{50} Mouse-oral 2000 mg/kg.[94]	The air concentration level of 1,2-DCB where irritation to humans begins (threshold of irritation), was found to be 0.15 mg/L[95]	-
26	**Solution-phase:** Sonication with1-pyrene carboxylic acid[27].	Single, few and multilayered graphene	<1.7×10^{-3}	~0.15 (532nm)	-	-	-
27	**Solution-phase:** Sonication with diisocyanates[28].	Few layers	1.6x10^{-3}	0.2-0.3 (532 nm), 0.11 in powder	LD_{50} Mouse-oral- 196 mg/kg. LD_{50} Rat-oral- 940 mg/kg.[96]	LD_{50} Rat-(male) dermal- 5000 uL/kg.[97] LD_{50} Rabbit skin- 7130 mg/kg.[96]	-
28	**Solution-phase:** ICl and IBr Intercalation[29].	2-3 layers	<1 x 10^{-3}	<0.2 (633 nm)	-	ICl Contact can severely irritate and burn the skin.[98]	ICl Can irritate the lungs, repeated exposure may cause bronchitis to develop with cough phlegm

							and shortness of breath.[98]
29	**Solution-phase:** Wet ball milling with N,N-dimethylformamide (DMF)[30].	≥3 layers	<7 x 10^{-4}	0.34	LD_{50} Oral - Rat - 2,800 mg/kg. LC_{50} Inhalation - Rat-4h - 9-15 mg/l.[62]	Skin – Human Result: Mild skin irritation - 24 h.[99]	-
30	**Solution-phase:** Sonication with Water:Acetone[31].	<5 layers	5x10^{-4}	0.25 (514 nm)	LD_{50} Rat-oral 9800 mg/kg. LD_{50} Mouse oral 3000 mg/kg. LD_{50} Rat-oral 10.7 mL/kg (=8450 mg/kg bw).[100]	LD50 Rabbit-dermal 20,000 mg/kg bw.[101]	Repeated exposure to 25-920 ppm: results chronic conjunctivitis, pharyngitis, bronchitis, gastritis, and gastroduodenitis. /Route not specified.[102]
31	**Solution-phase:** Sonication with quinquethiophene-terminated poly(ethylene glycol) (nonionic surfactant)[32].	2-3 layers	~6×10^{-3}	0.35	-	-	-
32	**Solution-phase:** Sonication with low boiling point solvents ($CHCl_3$and isopropanol)[33].	10 layers	3.1x10-4	< 0.4 (633 nm); starting powder 0.14	**$CHCl_3$:** LD_{50} Rabbit-oral- 9827 mg/kg. LD_{50} White rat-oral- 2180 mg/kg.[124]<br><br>**Isopropanol:** Refer column no. 13	**$CHCl_3$:** Skin and eye irritant Threshold of irritation: 20480 mg/cu m.[125]<br><br>**Isopropanol:** Refer column no. 13	**$CHCl_3$:**Carcinogenicity 2B - Group 2B: Possibly carcinogenic to humans.[126]<br><br>**Isopropanol:** Refer column no. 13
33	**Solution-phase:** Stirring with KC_8 in NMP[34].	Few layers	2x10^{-4}	Not given	-	-	-
34	**Solution-phase:** Sonication with hexadecyltrimethylammonium bromide/acetic acid[35].	Few layers	1.9x10^{-4}	~ 0.2 (532 nm)	LD_{50} Oral-Rat-410 mg/kg.[103]	Moderate skin irritation.[103]	

35	**Solution-phase:** Ultra sonication with perylenebisimide-Based Bolaamphiphile[36.]	Few layers and single layer	-	Estimated from spectra: 0.4-0.6 (532 nm)	-	-	-
36	**Solution-phase:** Sonication with surfactants [sodiumdodecylsulfate (SDS), dodecylbenzenesulfonic acid (SDBS), lithium dodecyl sulfate (LDS), cetyltrimethyl ammoniumbromide (CTAB), tetradecyltrimethylammonium bromide (TTAB), sodium cholate (SC), sodium deoxycholate (DOC), IGEPAL CO-890, Triton X-100, Tween 20 and Tween 80 sodiumtaurodeoxycholate (TDOC)][37].	1-16 layers	5×10^{-3}	0.25-0.6	**SDS** LD_{50}Oral-Rat-male and female-1,200 mg/kg.[104]<br><br>**SDBS** LD_{50} Oral-Rat-650 mg/kg.[105]<br><br>**CTAB** LD_{50}Oral-Rat-410 mg/kg.[106]<br><br>**TTAB** LD_{50}Oral-Rat-male and female-390 mg/kg.[107]<br><br>**SC**<br>-<br>**DOC** LD_{50}Oral-Rat-1,370 mg/kg. LD_{50}Oral-Mouse-1,050 mg/kg.[108]<br><br>**IGEPAL CO-890** LD_{50}Oral-Rat-4,000 mg/kg.[109]<br><br>**Triton X-100**<br>-<br>**Tween 20** LD_{50}Oral-Rat-40,554.0 mg/kg.[110]	**SDS** Skin irritant.[104]<br><br>**SDBS** Skin irritant.[105]<br><br>**CTAB** Moderate skin irritant.[106]<br><br>-<br><br>-<br>**DOC** LD_{50}Subcutaneous-Rat-2,430 mg/kg.[133]<br><br>**IGEPAL CO-890** Irritating to skin.[109]<br><br>-<br><br>**Tween 20** Mild skin irritation.[110]<br><br>**Tween 80**	

					**Tween 80** LD_{50}Oral-Mouse-25,000 mg/kg.[111]<br><br>**TDOC**<br>**-**	Mild skin irritation.[111]	
37	**Solution-phase:** Sonication with Tetraethylene glycol diacrylate[38].	Few layers	~3.9×10^{-3}	No data available	LD_{50}Oral-Rat-813 mg/kg.[112]	LD_{50}Dermal-Rabbit-> 3,000 mg/kg.[112]	
38	**Solution-phase:** Sonication of nanoribbons in hypophosphorous acid[39].	Multi layers	~3.3×10^{-3}	No data available	-	-	-
39	**Solution-phase:** Sonication with organosilanes (Phenyl triethoxysilane and 3-glycidoxypropyl trimethoxysilane)[40].	Few layers	3.3×10^{-3}	0.65 (532 nm)	**Phenyl triethoxysilane** LD_{50}Oral-Rat-2,734 mg/kg.[113]<br><br>**3-glycidoxypropyl trimethoxysilane** LD_{50}Oral-Rat-8,030 mg/kg.[114]	**Phenyl triethoxysilane** LD_{50}Dermal-Rabbit-3,167 mg/kg.[113]<br><br>**3-glycidoxypropyl trimethoxysilane** LD_{50}Dermal-Rabbit-4,248 mg/kg.[114]	-
40	**Solution-phase:** Microwave assisted exfoliation of $FeCl_3$ and CH_3 NO_2 co-intercalated graphite.[41]	Multilayers	1.6×10^{-3}	0 but layer graphenes (~5)	**$FeCl_3$** LD_{50}Oral-Mouse-1,300 mg/kg.[115]<br><br>**CH_3 NO_2** LD_{50}Oral-Rat-male and female-1,478 mg/kg.[115]	**$FeCl_3$** LD_{50}Dermal-Rabbit-> 2,000 mg/kg.[115]<br><br>**-**	

41	**<u>Solution-phase:</u>** Sonication with N-Methylpyrrolidone[42].	Single, bi and few layers/few layers/few layers (<5)/~3-4 layers/monolayer (7-12 wt.%)	~1.2×10^{-3}/ 1.3×10^{-3}/~9×10^{-4}/1.5×10^{-3}/2×10^{-4}	<0.25 for bath, <0.35 for tip (633 nm)/0.25-2.5 (457, 514, 633 nm)/~0.36 /<0.5 (633 nm)/0 is thick films and 0.2 is for thin films	LD_{50}Oral-Rat-3,914 mg/kg.[59]	LD_{50} Dermal-Rabbit-8,000 mg/kg.[59]	-
42	**<u>Solution-phase:</u>** Sonication with surfactants (sodium cholate hydrate, didodecyldimethylammonium bromide, and Triton X-100)[43].	Few layers	1.2×10^{-3}	0.45 (514 nm)	-	-	-
43	**<u>Solution-phase:</u>** Sonication with non-ionic block copolymers (Pluronics and Tetronics)[44].	1 to ~10 layers	1.1×10^{-3}	0.9 (514 nm)	-	-	-
44	**<u>Solution-phase:</u>** Exfoliation in ionic liquid (1-butyl-3-methyl-imidazolium bis(trifluoromethanesulfonyl)imide)[45].	Single, bi, few and multi layers	9.5×10^{-4}	0.17	LD_{50}Oral-Rat-> 50 -< 300 mg/kg.[117]	-	May cause damage to organs through prolonged or repeated exposure.[117]
45	**<u>Solution-phase:</u>** Sonication with sodium cholate[46].	1-10 layers (20% is monolayer)	3×10^{-4}	0.57 (633 nm)	-	-	-
46	**<u>Solution-phase:</u>** Graphene sheets were made from natural graphite	Few layers	1.4×10^{-5} (chemical exfoliation) and 5×10^{-4}	1 (633 nm)	**DMF** LD_{50} Oral-Rat-2,800 mg/kg. LC_{50} Inhalation-	**DMF** Mild skin irritation.[99]	-

	flakes, intercalated by oleum And tetrabutylammoniumcations, and suspended in N,N-dimethylformamide (DMF) followed by solvothermal reduction process[47].		(reduction)		Rat-4 h - 9-15 mg/l.[99]		
47	<u>**Solution-phase:**</u> Sonication with dihydroxy sodiumdeoxycholate (DOC)[48].	1-3 layers (60% are single layer)	~7.6×10^{-6}	Not given	**DOC** LD_{50} Oral-Rat-1,370 mg/kg. LD_{50} Oral-Mouse-1,050 mg/kg.[108]	**DOC** LD_{50} Subcutaneous-Rat-2,430 mg/kg.[108]	-
48	<u>**Solution-phase:**</u> Heating (at 1000 °C, 3% H_2 in Ar) followed by sonication in 1,2-dichloroethane (DCE) solution of poly(m-phenylenevinylene-co-2,5-dioctoxy-p-phenylenevinylene)[49].	Few layers (mostly ≤3 layers)	-	Spectrum is poor and cannot be detected	**DCE** LD_{50}Oral-Rat-670.0 mg/kg.[118]	**DCE** LD_{50}Dermal-Rabbit-2,800 mg/kg.[118]	-
49	<u>**Solution-phase:**</u> Thermal treatment of graphite with liquid crystal (1,10 -didodecyl-4,40 -bipyridiniumbis(triflimide)[50].	15, 30, 37 and 18% of the GNSs comprised 2- 3, 4, 5–10 and >10 layers of graphene, respectively.	-	0 (633 nm)	-	-	-

50	**Solution-phase:** Electrochemical exfoliation with LiCl/propylene carbonate[51].	< 5 layers	~0.25	0 (514)	**LiCl**<br>-<br>**Propylene carbonate**<br>LD_{50} Oral-Rat-> 5,000 mg/kg.[119]	-<br>**Propylene carbonate**<br>LD_{50}Dermal-Rabbit-> 2,000 mg/kg.[119]	-<br>-
51	**Solution-phase:** K(THF)$_x$C24 (THF = tetrahydrofuran, *x* = 1-3)[x52].	Multi layers	-	>1	-	-	-
52	**Solution-phase:** Methods produced through GO (e.g. Concentrated Sulfuric acid (H_2SO_4) (a) in combination with fuming Nitric acid (HNO_3) and Potassium chlorate ($KClO_3$) (Staudenmaier method) or (b) in combination with concentrated HNO_3 and $KClO_3$ (Hofmann method) or (c) in the absence of HNO_3 but in the presence of Sodium nitrate ($NaNO_3$) and Potassium permanganate ($KMnO_4$) (Hummers method)[53].	-	≤0.45	0.9 - ≥1	**H_2SO_4**<br>LD_{50} Oral-Rat-2,140 mg/kg.[120]<br>**HNO_3**<br>-<br>**$KClO_3$**<br>LD50 Oral-Rat-1,870 mg/kg.[121]<br>**$NaNO_3$**<br>LD_{50} Oral-Rat-3,430 mg/kg.[122]<br>**$KMnO_4$**<br>LD50 Oral-Rat-1,090 mg/kg.[123]	**H_2SO_4**<br>Extremely corrosive and destructive to tissue.[120]<br>**HNO_3**<br>-<br>**$KClO_3$**<br>LD50 Dermal-Rat-male and female- > 2,000 mg/kg.[121]<br>**$NaNO_3$**<br>LD_{50} Dermal-Rat- > 5,000 mg/kg.[122]<br>**$KMnO_4$**<br>Corrosive.[123]	

53	**<u>Solution and Solid-phase</u>** Dry ball milling graphite in the presence of hydrogen, carbon dioxide, sulfur trioxide, or carbon dioxide/sulfur trioxide mixture[128].	Few layers (Edge functionalized)	0.1	0.79-1.44	**SO_3** LC50Inhalation-Rat-4 h-375 mg/m3 Extremely corrosive and destructive to tissue.[129]	**SO_3** Severe skin irritant.[129]	
54	**<u>Solution Phase</u>** Graphite sonicated with the bio based solvent cyrene for 15 minutes and then centrifuged to obtain graphene oxide[130].	Mono to few layer graphene (less than 10)	0.06	0.20	LD50Oral-Rat-female-> 2,000 mg/kg[131].	No skin irritation	-

S.6 Computational studies on interaction of graphene with curcumin

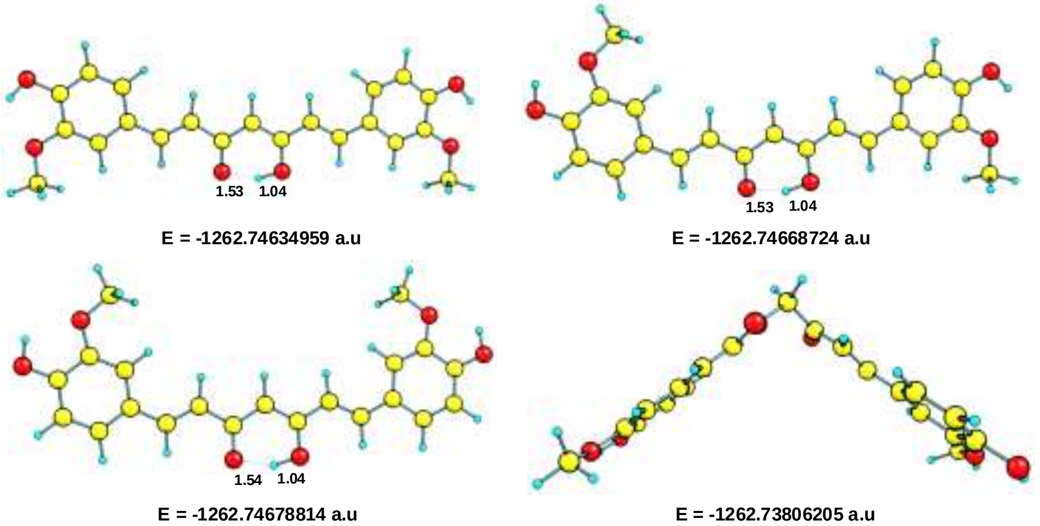

Figure S13: The optimized structures of curcumin conformers with their energies (a.u) at B97-D/6-31G** level of theory.

Curcumin conformers and curcumin-demethoxyderivatives

Curcumin (keto-enolic form-1)

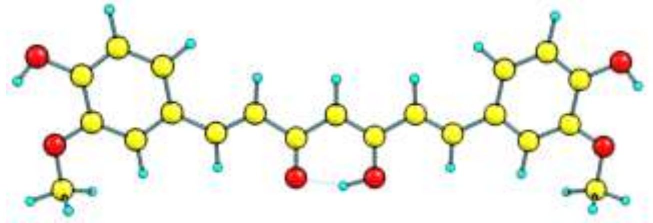

Curcumin (keto-enolic form -2)

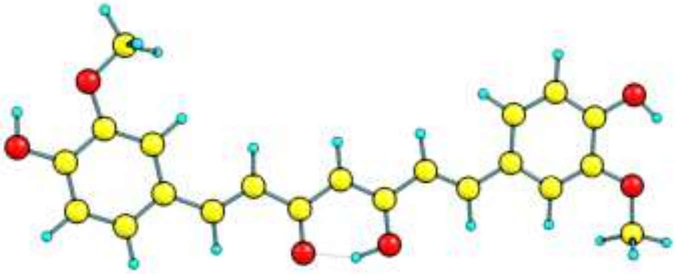

Standard orientation:

Curcumin (keto-enolic form -3)

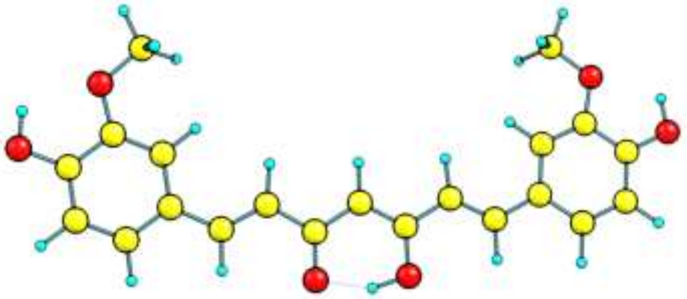

Curcumin (di-keto form)

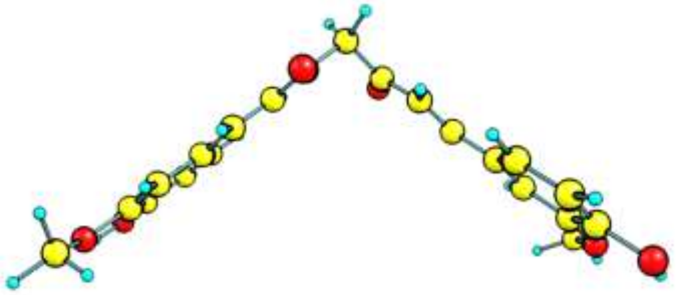

DemethoxyCurcumin

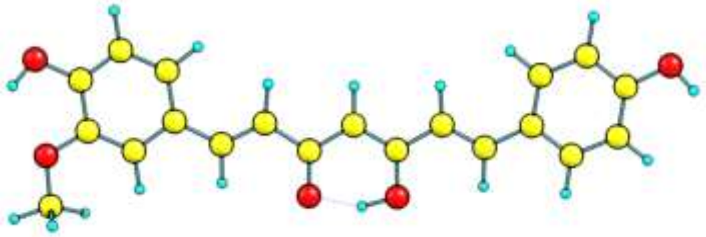

Bis-demethoxyCurcumin

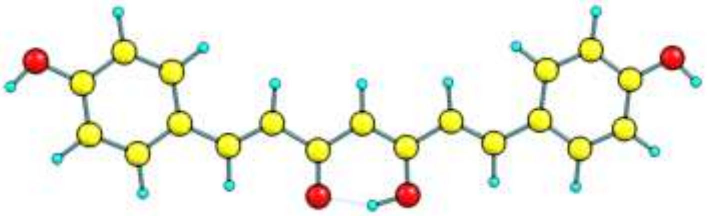

Graphene models

$C_{160}H_{32}$

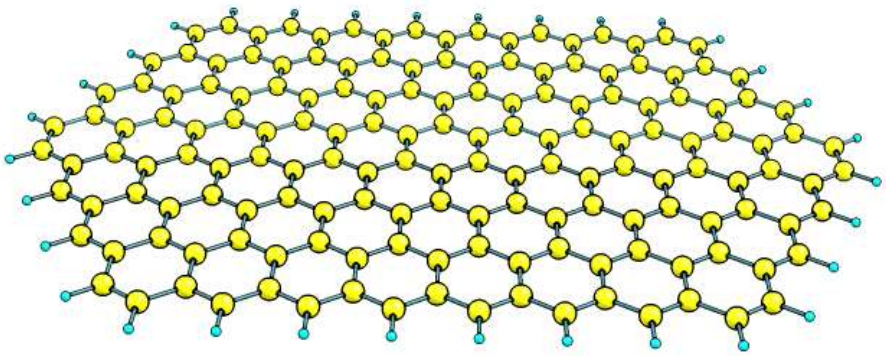

$C_{168}H_{36}$

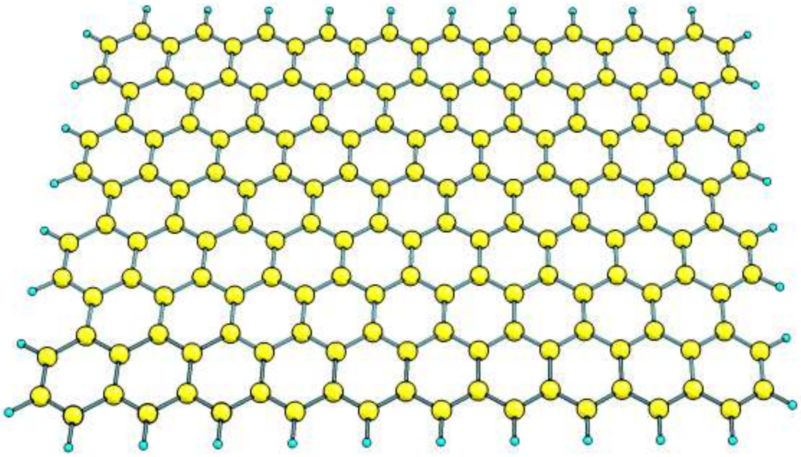

$C_{156}H_{36}$

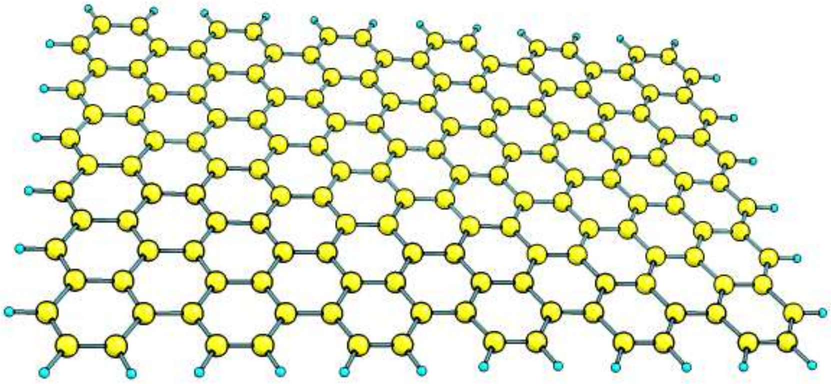

$C_{160}H_{32}$/curcumin

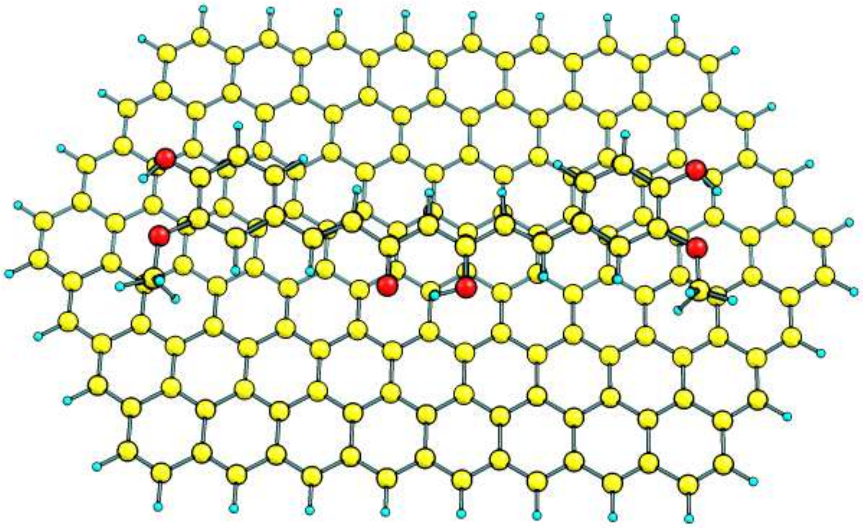

$C_{160}H_{32}$/demethoxycurcumin

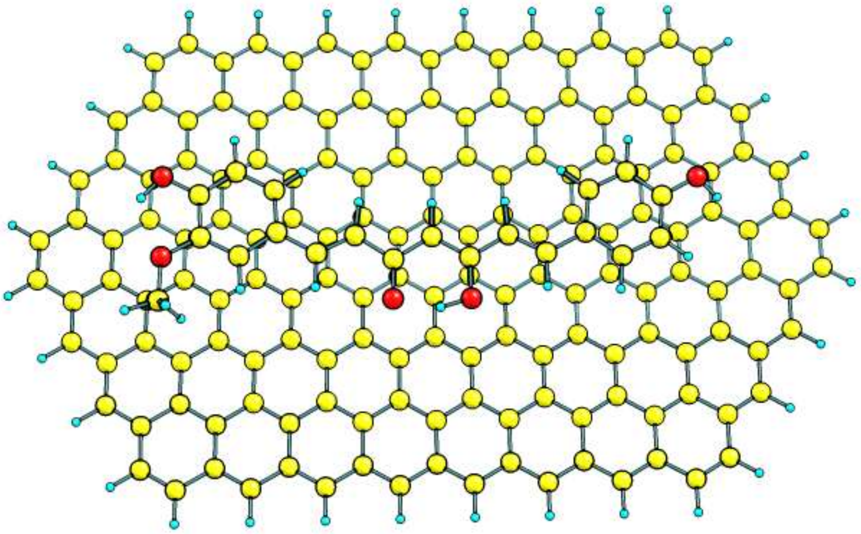

$C_{160}H_{32}$/bis-demethoxycurcumin

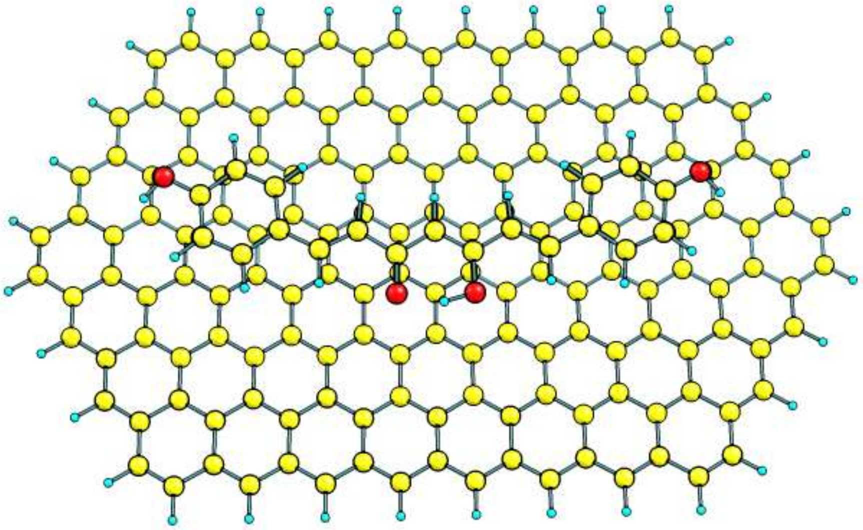

$C_{168}H_{36}$/curcumin

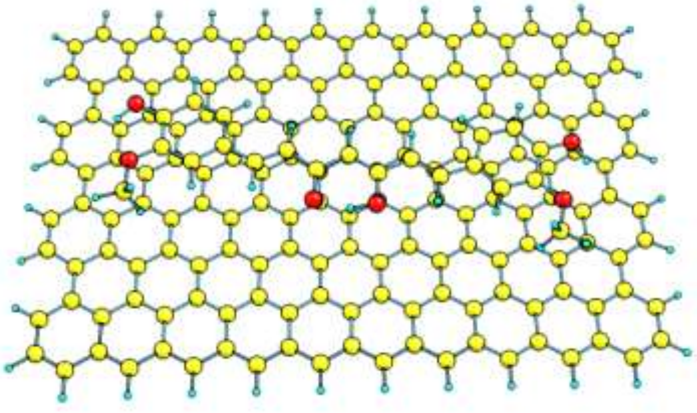

$C_{156}H_{36}$/curcumin

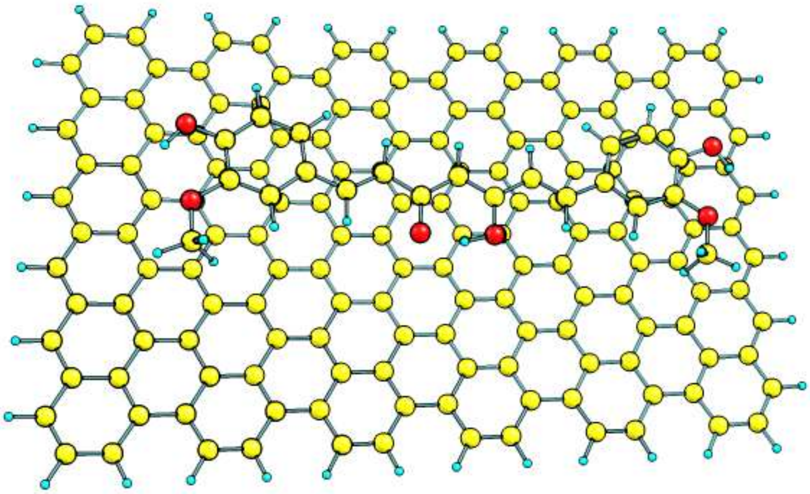

S7. Production of few layer graphene- NR thin film nano-composite

(a) **Preparation of graphene dispersion:** Milled Graphite:Curcumin:Darvan (1:3:0.125) sample was made into a 30 wt. % aqueous dispersion by using probe sonication in distilled water (2 min. at 25 % amplitude).

(b) **Preparation of graphene - NR latex mixture:** The above graphene dispersion (at 0.3, 0.7, 1.5, 3 and 5 phr) was then added to the compounded NR latex using probe sonication technique (3 min. at 20 % amplitude).

(c) **Regulation of compound:** The graphene- NR Latex was stabilized by addition of 1% ammonia and had a solids content of 48 ±3 %. The mixture was stirred slowly to ensure homogeneity and the optimum cure was checked using the following chloroform test.

In a 50 mL beaker 5 mL of the above made NR latex was transferred. Then, 5 mL of chloroform was added to the same and stirred the mixture gently and continuously to obtain a coagulum. This mixture was then kept between two filter papers and pressed. This solid material was broken off gently and judged the nature of cure. All the nanocomposites were made from normal cure NR latex.

Graphene incorporated natural rubber nanocomposite thin films were produced using the following steps;

(1) Pre-treatment of glass molds

(2) Latex dipping

(3) Vulcanization followed by stripping the thin film from mould using silica powder.

Prior to each dipping procedure cleaning of glass molds were done by brushing with detergent water followed by washing with hot water. Later, the molds were dried in hot air oven at 70 $^{\circ}$C. For lab scale production, we have used a semi-automatic type dipping machine supplied by PLASTOMEK Private Ltd. India. The thickness of the produced nanocomposite thin film samples can be controlled by varying the dipping speed.

Subsequent to each dipping, the skim and cream on the NR latex surface were removed using a cloth or sponge. The presence of any bubbles was also removed in order to avoid any weak spots on the thin films. The mold fitted on machine is then dipped slowly into the NR latex at a rate of speed of dipping from 1-1.5 cm/sec. After immersing mold up to a required length, the mold was withdrawn slowly and rotated the mold to ensure a uniform flow of the NR Latex. Then, the mold was kept in hot air oven for drying at 70 $^{\circ}$C for 2-3 min. After drying and cooling, a second dipping was done and kept for drying again at 70 $^{\circ}$C for 2-3 min. For vulcanization, the thin films on the mold are transferred to a hot air oven and heated the product for about 45 min. at a temperature of 80 ± 5 $^{\circ}$C.

S8 Characterization of few layer graphene-NR thin film nano-composite

S8.1 TEM analysis

Morphology of the few layer graphene NR thin film nano-composites was analyzed using a JEOL JEM-2010TEM at 200 kV. Samples were prepared by cryo-microtoming at -70 ºC.

S8.1.1 TEM analysis of Graphite:Curcumin:Darvan-NR thin film nanocomposite

The TEM images of 1.5 phr Graphite:Curcumin:Darvan containing NR thin film nano-composite are shown as Figure S14. The samples show a network like structure.

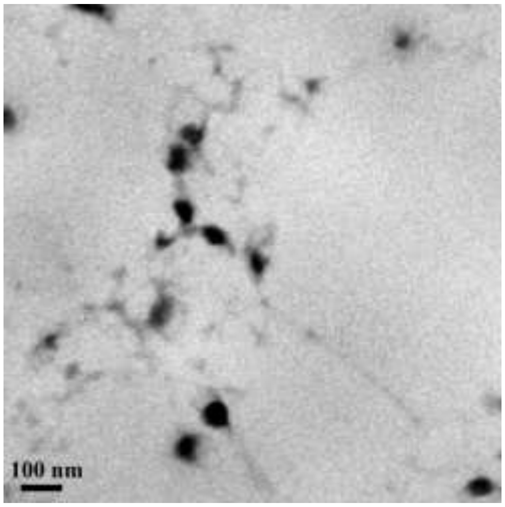

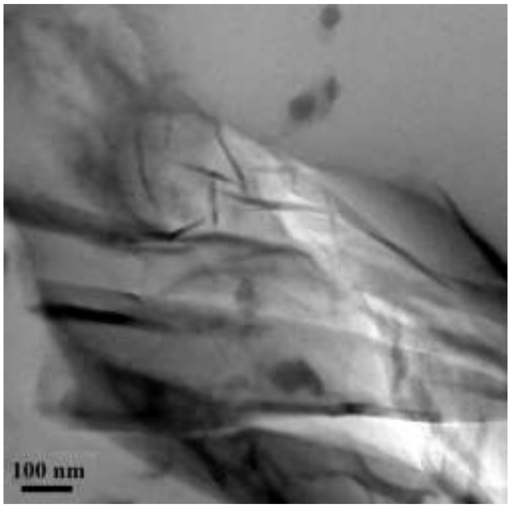

Figure S14 TEM images of 1.5 phr graphene (Graphite:Curcumin:Darvan) reinforced NR latex thin films.

S8.2 Stability of curcumin under the processing condition

1.5 phr few layer graphene -NR thin film nano-composite weighing about 1.11 g was Soxhlet extracted using200 mL of dichloromethane for 2 h. After completion of extraction, a greenish orange color extract was obtained which was then cooled, concentrated using rotavapor to get a greenish orange precipitate.

Characterization of curcumin (obtained after Soxhlet extraction) by thin layer chromatography:

The extract obtained after extraction was subjected to TLC using pre-coated silica gel (Merck 60 F_{254}, 20cm×20cm) plates. The curcumin was separated using chloroform: methanol: (9.5:0.5. (Figure S15).

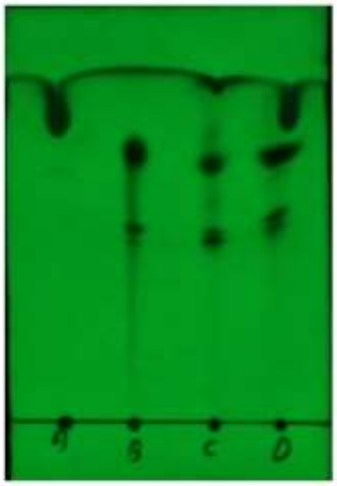

Figure S15: The TLC results of dichloromethane extract obtained after Soxhlet extraction.

* Where A = Soxhlet extract from natural rubber latex (control sample)

B = Commercial sample of curcumin

C = Soxhlet extract from control curcumin (thin film having 0.7 phr curcumin and no graphene)

D = Soxhlet extract from 1.5 phr graphene (Graphene/Curcumin/Darwan)/-NR thin film nanocomposite

A commercial sample of curcumin (B) exhibits three spots (R_f values 0.78, 0.54, 0.50). Similar spots are obtained from control curcumin (C) and 1.5 phr graphene (dry milled Graphene/Curcumin/Darwan)/-NR thin film nano-composite (D). The results confirm that the leached out extract from both control curcumin (C) and 1.5 phr graphene (D) is curcumin. Thus the process does not result in any degradation of curcumin.

Characterization of curcumin (obtained after Soxhlet extraction) by High Performance Liquid Chromatography (HPLC):

The HPLC system consisting of Agilent 1260 series PDA detector was used for this study. Chromatographic separation was achieved using Agilent RP-C_{18} column (4.6mm×150mm, 5μm). The mobile phase comprised of solvent A: water (1% acetic acid) which was adjusted to pH of 3.0 using 50 % triethanolamine and solvent B: Acetonitrile in the ratio of 50:50 v/v. The isocratic elution was carried out with the flow rate of 1.5 ml/min at ambient temperature. Prior to use, all the samples were filtered through a 0.45 μm membrane filter. The chromatogram was obtained at wavelength of 254 nm.

Preparation of standard and different sample solutions for HPLC:

Stock solutions of standard curcumin, dichloromethane extracts obtained after Soxhlet extraction of natural rubber thin film, curcumin incorporated natural rubber thin film and 1.5phr few layer graphene natural rubber nanocomposite thin film samples were prepared by dissolving 10mg of sample in methanol. The observed peaks (Figure S16) are in agreement with the HPLC chromatograms of standard curcumin as reported by previous researchers[131].

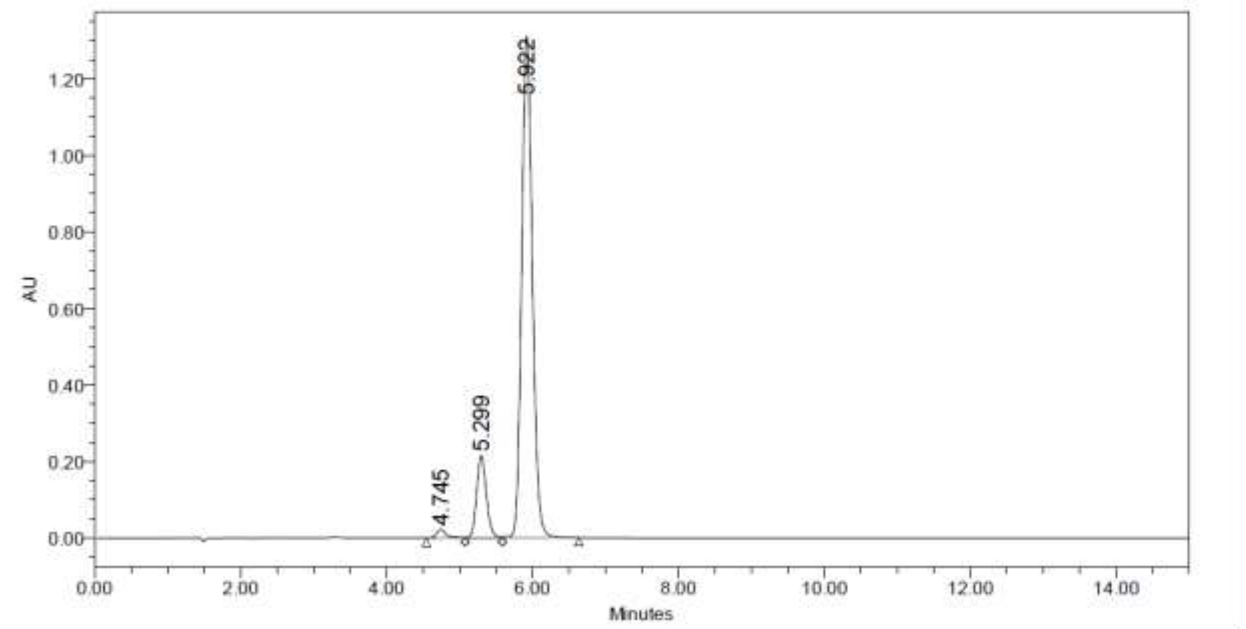

Figure S16: HPLC chromatogram of Soxhlet extract obtained from 1.5 phr Graphite:Curcumin:Darvan thin film sample.

S9 Biocompatibility of graphene-NR latex thin film nanocomposite

Table S5. Qualitative grading scheme for scoring cytotoxicity based on morphology of cells (Balb/c3T3).

Grade	Reactivity	Conditions of all culture
0	None	Discrete intracytoplasmatic granules, no cell lysis, no reduction of cell growth
1	Slight	Not more than 20% of the cells are round, loosely attached and without intracytoplasmic granules, or show changes in morphology; occasional lysed cells are present; only slight growth inhibition observable
2	Mild	Not more than 50% of the cells are round, devoid of intracytoplasmic granules, no extensive cell lysis; not more than 50% growth inhibition observable
3	Moderate	Not more than 70% of the cell layers contain rounded cells or are lysed; cell layers not completely destroyed, but more than 50% growth inhibition observable
4	Severe	Nearly complete or complete destruction of the cell layers

Table S6: Primary index used to predict the skin toxicity on New Zealand white Rabbits.

Mean Score	Response category
0 to 0.4	Negligible
0.5 to 1.9	Slight
2 to 4.9	Moderate
5 to 8	Severe

Table S7: Experimental procedure used for performing skin sensitization on guinea pigs.

Group No.	Animal No.	Sex	Treatment Group	Intradermal Induction Phase (0.1 mL)			Topical induction phase (0.5 mL using a patch)*		Challenge phase # (0.5 mL using a patch)*
				Injection I	Injection II	Injection III	10% SLS	Treatment	
G1	1-5	F	Polar solvent control	1: 1 mixture (v/v) FCA + (saline)	Polar solvent alone	50% w/v formulation of the vehicle in a 1:l mixture (v/v) FCA + (saline)	Yes	Polar solvent	Polar solvent & Polar extract of Test item
G2	6-15	F	Test item in polar solvent	1: 1 mixture (v/v) FCA + (saline)	Test item in polar solvent	Polar extract of Test item in a 1:1 mixture (v/v) FCA + (saline)	Yes	Polar extract of Test item	Polar solvent & Polar extract of Test item
G3	16-20	F	Non polar solvent control	1: 1 mixture (v/v) FCA + (sunflower oil)	Non polar solvent alone	50% w/v formulation of the vehicle in a 1:l mixture (v/v) FCA + (sunflower oil)	Yes	Non polar solvent	Non polar solvent & Non polar extract of Test item
G4	21-30	F	Test item in non polar solvent	1: 1 mixture (v/v) FCA + (sunflower oil)	Test item in non polar solvent	Non polar extract of Test item in a 1:1 mixture (v/v) FCA + (sunflower oil)	Yes	Non polar extract of Test item	Non polar solvent & Non polar extract of Test item

F- Female; FCA - Freund's Complete Adjuvant; SLS - Sodium Lauryl Sulphate;
* Patch area = 8 cm^2 approximately;
# Gauze soaked in the respective preparation
Intradermal Injection was given on Day 0 at sites A, B and C
Topical application was applied on Day 7
Challenge dose was applied on Day 21
Sites A, B and C are shown below:

Figure S17: 1 – Cranial end; 2 – 0.1 ml intradermal injection sites; 3 – clipped intrascapular region; 4 – Caudal end

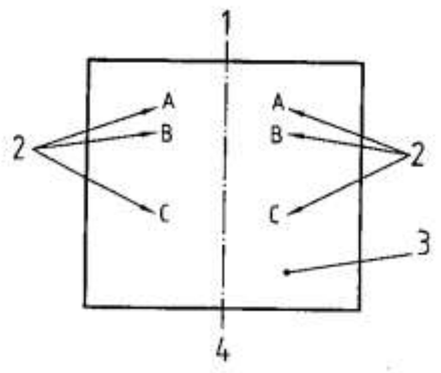

Table S8. Positive control experiments performed with α-Hexylcinnamaldehyde for skin sensitization on guinea pigs.

Concentration of α-Hexylcinnamaldehyde			Vehicle used	Result	
**Induction I**	**Induction 2**	**Challenge**		**No of animals +ve**	**Maximum reaction grading**
0.5% v/v	50% v/v	10% v/v	4:1 v/v acetone: sunflower oil	+ve in 7/10 animals	Grade 2 - Moderate and confluent erythema

Table S9. Primary index used to predict for skin sensitization on guinea pigs.

**Patch test reaction**	**Grading scale**
No visible change	0
Discrete or patchy erythema	1
Moderate and confluent erythema	2
Intense erythema and/or swelling	3

References

1. V. León, A.M. Rodriguez, P. Prieto, M. Prato, E. Vázquez, Exfoliation of Graphite with Triazine Derivatives under Ball-Milling Conditions: Preparation of Few-Layer Graphene via Selective Noncovalent Interactions, ACS Nano. 8 (2014) 563–571. doi:10.1021/nn405148t.
2. A.C. Ferrari, D.M. Basko, Raman spectroscopy as a versatile tool for studying the properties of graphene, Nat Nano. 8 (2013) 235–246. http://dx.doi.org/10.1038/nnano.2013.46.
3. L. Cancado, A. Reina, J. Kong, M.S. Dresselhaus, Geometrical approach for the study of G′ band in the Raman spectrum of monolayer graphene, bilayer graphene, and bulk graphite, Phys. Rev. B. 77 (2008) 245408. doi:10.1103/PhysRevB.77.245408.
4. V. Leon, M. Quintana, M.A. Herrero, J.L.G. Fierro, A. de la Hoz, M. Prato, E. Vazquez, Few-layer graphenes from ball-milling of graphite with melamine, Chem. Commun. 47 (2011) 10936–10938. doi:10.1039/C1CC14595A.
5. K.R. Paton, E. Varrla, C. Backes, R.J. Smith, U. Khan, A. O'Neill et al., Scalable production of large quantities of defect-free few-layer graphene by shear exfoliation in liquids, Nat Mater. 13 (2014) 624–630. http://dx.doi.org/10.1038/nmat3944.
6. George, G. et al. Thermally conductive thin films derived from defect free graphene-natural rubber latex nanocomposite: Preparation and properties. Carbon 119, (527-534), 2017.
7. Knieke, C. et al. Scalable production of graphene sheets by mechanical delamination. *Carbon***48**, 3196-3204, (2010).
8. Herron, C. R., Coleman, K. S., Edwards, R. S. &Mendis, B. G. Simple and scalable route for the 'bottom-up' synthesis of few-layer graphene platelets and thin films. *J. Mater. Chem.* **21**, 3378- 3383, (2011).
9. Viculis, L. M., Mack, J. J., Mayer, O. M., Hahn, H. T. &Kaner, R. B. Intercalation and exfoliation routes to graphite nanoplatelets. *J. Mater. Chem.* **15**, 974-978, (2005).
10. Liao, K.-H. *et al.* Aqueous Only Route toward Graphene from Graphite Oxide. *ACS Nano* **5**, 1253-1258, (2011).
11. Feng, H., Cheng, R., Zhao, X., Duan, X. & Li, J. A low-temperature method to produce highly reduced graphene oxide. *Nat Commun***4**, 1539, (2013).
12. Lu, W. *et al.* High-yield, large-scale production of few-layer graphene flakes within seconds: using chlorosulfonic acid and H_2O_2 as exfoliating agents. *J. Mater. Chem.* **22**, 8775-8777, (2012).
13. Parviz, D. *et al.* Dispersions of Non-Covalently Functionalized Graphene with Minimal Stabilizer. *ACS Nano* **6**, 8857-8867, (2012).
14. Eun-Young, C., Won San, C., Young Boo, L. & Yong-Young, N. Production of graphene by exfoliation of graphite in a volatile organic solvent. *Nanotechnology* **22**, 365601, (2011).
15. Green, A. A. &Hersam, M. C. Solution Phase Production of Graphene with Controlled Thickness via Density Differentiation. *Nano Lett.*, (2009).
16. Oh, S. Y., Kim, S. H., Chi, Y. S. & Kang, T. J. Fabrication of oxide-free graphene suspension and transparent thin films using amide solvent and thermal treatment. *Appl. Surf. Sci.* **258**, 8837-8844, (2012).
17. Park, K. H. *et al.* Exfoliation of Non-Oxidized Graphene Flakes for Scalable Conductive Film. *Nano Lett.* **12**, 2871-2876, (2012).

18. Chabot, V., Kim, B., Sloper, B., Tzoganakis, C. & Yu, A. High yield production and purification of few layer graphene by Gum Arabic assisted physical sonication. *Sci. Rep.* **3**, 1378, (2013).
19. Shang, N. G. *et al.* Controllable selective exfoliation of high-quality graphenenanosheets and nanodots by ionic liquid assisted grinding. *Chem. Commun. (Cambridge, U. K.)* **48**, 1877-1879, (2012).
20. (a) Behabtu, N. *et al.* Spontaneous high-concentration dispersions and liquid crystals of graphene. *Nat Nano* **5**, 406-411, (2010).(b) Lu, W. et al. High-yield, large-scale production of few-layer graphene flakes within seconds: using chlorosulfonic acid and H2O2 as exfoliating agents. J. Mater. Chem. 22, 8775-8777, (2012).
21. Yi, M., Shen, Z., Zhang, X. & Ma, S. Vessel diameter and liquid height dependent sonication-assisted production of few-layer graphene. *J. Mater. Sci.* **47**, 8234-8244, (2012).
22. Lotya, M. *et al.* Liquid Phase Production of Graphene by Exfoliation of Graphite in Surfactant/Water Solutions. *J. Am. Chem. Soc.* **131**, 3611-3620, (2009).
23. Sanna, R. *et al.* Synthesis and characterization of graphene-containing thermoresponsivenanocomposite hydrogels of poly(N-vinylcaprolactam) prepared by frontal polymerization. *Journal of Polymer Science Part A: Polymer Chemistry* **50**, 4110-4118, (2012).
24. Nuvoli, D. *et al.* High concentration few-layer graphene sheets obtained by liquid phase exfoliation of graphite in ionic liquid. *J. Mater. Chem.* **21**, 3428-3431, (2011).
25. Shahil, K. M. F. &Balandin, A. A. Graphene–Multilayer Graphene Nanocomposites as Highly Efficient Thermal Interface Materials. *Nano Lett.* **12**, 861-867, (2012).
26. Hamilton, C. E., Lomeda, J. R., Sun, Z., Tour, J. M. & Barron, A. R. High-Yield Organic Dispersions of UnfunctionalizedGraphene. *Nano Lett.* **9**, 3460-3462, (2009).
27. An, X., Simmons, T., Shah, R., Wolfe, C., Lewis, K.M., Washington, M., et al. *Nano Lett.***10,** 4295–4301, (2010).
28. Scognamillo, S. *et al.* Synthesis and characterization of nanocomposites of thermoplastic polyurethane with both graphene and graphene nanoribbon fillers. *Polymer* **53**, 4019-4024, (2012).
29. Shih, C.-J. *et al.* Bi- and trilayergraphene solutions. *Nat Nano* **6**, 439-445, (2011)
30. Zhao, W. et al. Preparation of graphene by exfoliation of graphite using wet ball milling. *J. Mater. Chem*. **20**, 5817-5819, (2010).
31. Min, Y., Zhigang, S., Xiaojing, Z. &Shulin, M. Achieving concentrated graphene dispersions in water/acetone mixtures by the strategy of tailoring Hansen solubility parameters. *J. Phys. D: Appl. Phys.***46**, 025301, (2013).
32. Kang, M. S., Kim, K. T., Lee, J. U. & Jo, W. H. Direct exfoliation of graphite using a non-ionic polymer surfactant for fabrication of transparent and conductive graphene films. *Journal of Materials Chemistry C* **1**, 1870-1875, (2013).
33. O'Neill, A., Khan, U., Nirmalraj, P. N., Boland, J. & Coleman, J. N. Graphene Dispersion and Exfoliation in Low Boiling Point Solvents. *Journal of Physical Chemistry C* **115**, 5422-5428, (2011).
34. Catheline, A. *et al.* Graphene solutions. *Chem. Commun. (Cambridge, U. K.)* **47**, 5470-5472, (2011).

35. Vadukumpully, S., Paul, J. &Valiyaveettil, S. Cationic surfactant mediated exfoliation of graphite into graphene flakes. *Carbon* **47**, 3288-3294, (2009).
36. Englert, J. M. *et al.* Soluble Graphene: Generation of Aqueous Graphene Solutions Aided by a Perylenebisimide-Based Bolaamphiphile. *Adv. Mater. (Weinheim, Ger.)* **21**, 4265-4269, (2009).
37. Smith, R. J., Lotya, M. & Coleman, J. N. The importance of repulsive potential barriers for the dispersion of graphene using surfactants. *New Journal of Physics* **12**, (2010).
38. Alzari, V. *et al.* In situ production of high filler content graphene-based polymer nanocomposites by reactive processing. *J. Mater. Chem.* **21**, 16544-16549, (2011).
39. Nuvoli, D. *et al.* The production of concentrated dispersions of few-layer graphene by the direct exfoliation of graphite in organosilanes. *Nanoscale Research Letters* **7**, 674, (2012).
40. Nuvoli, D. *et al.* The production of concentrated dispersions of few-layer graphene by the direct exfoliation of graphite in organosilanes. *Nanoscale Research Letters* **7**, 674, (2012).
41. Fu, W., Kiggans, J., Overbury, S. H., Schwartz, V. & Liang, C. Low-temperature exfoliation of multilayer-graphene material from FeCl3 and CH3NO2 co-intercalated graphite compound. *Chem. Commun. (Cambridge, U. K.)* **47**, 5265-5267, (2011).
42. (a) Torrisi, F. et al. Inkjet-Printed Graphene Electronics. *ACS Nano***6**, 2992-3006, (2012). (b)Khan, U. *et al.* Size selection of dispersed, exfoliated graphene flakes by controlled centrifugation. *Carbon* **50**, 470-475, (2012). (c) Alzari, V. *et al.* Graphene-containing thermoresponsivenanocomposite hydrogels of poly(N-isopropylacrylamide) prepared by frontal polymerization. *J. Mater. Chem.* **21**, 8727-8733, (2011). (d) Khan, U. *et al.* Solvent-Exfoliated Graphene at Extremely High Concentration. *Langmuir* **27**, 9077-9082, (2011). (e)
43. Buzaglo, M. *et al.* Critical parameters in exfoliating graphite into graphene. *Phys. Chem. Chem. Phys.* **15**, 4428-4435, (2013).
44. Seo, J.-W. T., Green, A. A., Antaris, A. L. &Hersam, M. C. High-Concentration Aqueous Dispersions of Graphene Using Nonionic, Biocompatible Block Copolymers. *The Journal of Physical Chemistry Letters* **2**, 1004-1008, (2011).
45. Wang, X. Q. *et al.* Direct exfoliation of natural graphite into micrometre size few layers graphene sheets using ionic liquids. *Chem. Commun. (Cambridge, U. K.)* **46**, 4487-4489, (2010).
46. Lotya, M., King, P. J., Khan, U., De, S. & Coleman, J. N. High-Concentration, Surfactant- Stabilized Graphene Dispersions. *ACS Nano* **4**, 3155-3162, (2010).
47. Wang, H., Robinson, J. T., Li, X. & Dai, H. Solvothermal Reduction of Chemically Exfoliated Graphene Sheets. *J. Am. Chem. Soc.* **131**, 9910-9911, (2009).
48. Marago, O. M. *et al.* Brownian Motion of Graphene. *ACS Nano* **4**, 7515-7523, (2010).
49. Li, X. L., Wang, X. R., Zhang, L., Lee, S. W. & Dai, H. J. Chemically derived, ultrasmoothgraphenenanoribbon semiconductors. *Science* **319**, 1229-1232, (2008).
50. Safavi, A., Tohidi, M., Mahyari, F. A. &Shahbaazi, H. One-pot synthesis of large scale graphenenanosheets from graphite-liquid crystal composite via thermal treatment. *J. Mater. Chem.* **22**, 3825-3831, (2012).
51. Wang, J., Manga, K. K., Bao, Q. &Loh, K. P. High-Yield Synthesis of Few-Layer Graphene Flakes through Electrochemical Expansion of Graphite in Propylene Carbonate Electrolyte. *J. Am. Chem. Soc.* **133**, 8888-8891, (2011).

52. Valles, C. *et al.* Solutions of Negatively Charged Graphene Sheets and Ribbons. *J. Am. Chem. Soc.* **130**, 15802-+, (2008).
53. Poh, H.L., Sanek, F., Ambrosi, A., Zhao, G., Sofer, Z.,&Pumera, M. Graphenes prepared by Staudenmaier, Hofmann and Hummers methods with consequent thermal exfoliation exhibit very different electrochemical properties. *Nanoscale***4**, 3515-3522, (2012).
54. Curcumin; MSDS Product No. C7727; Available from Sigma-Aldrich http://www.sigmaaldrich.com/MSDS/MSDS/DisplayMSDSPage.do?country=IN&language=en&productNumber=C7727&brand=SIGMA&PageToGoToURL=http%3A%2F%2Fwww.sigmaaldrich.com%2Fcatalog%2Fproduct%2Fsigma%2Fc7727%3Flang%3Den. (Accessed on 07.04.2017)
55. Kohli K, Ali J, Ansari M J, Raheman Z. Curcumin: A natural antiinflammatory agent. Indian J Pharmacol 2005;37:141-7
56. Sittisomwong N., Leelasangaluk V., Chivapat S., Wangmad A., Ragsaman P., Chuntarachaya C., Acute and subchronic toxicity of turmeric. Bull. Dept. Med. Sci. 1990. 32(3):101-111.
57. Tetrahydrocurcumin; http://www.chemblink.com/MSDS/MSDSFiles/36062-04-1_Clear%20Synth.pdf(Accessed on 02.05.2017).
58. Quercetin; MSDS Product No. Q4951; Available from Sigma-Aldrich http://www.sigmaaldrich.com/MSDS/MSDS/DisplayMSDSPage.do?country=IN&language=en&productNumber=Q4951&brand=SIGMA&PageToGoToURL=http%3A%2F%2Fwww.sigmaaldrich.com%2Fcatalog%2Fsearch%3Fterm%3DQuercetin%26interface%3DProduct%2520Name%26N%3D0%2B%26mode%3Dmode%2520matchpartialmax%26lang%3Den%26region%3DIN%26focus%3DproductN%3D0%2520220003048%2520219853286%2520219853147.(Accessed on 27.04.2017)
59. 1-Methyl-2-pyrrolidinone; MSDS Product No.M6762; Available from Sigma Aldrich.http://www.sigmaaldrich.com/MSDS/MSDS/DisplayMSDSPage.do?country=IN&language=en&productNumber=M6762&brand=SIGMA&PageToGoToURL=%252Fcatalog%252Fproduct%252Fsigma%252Fm6762%253Flang%253Den(Accessed on 26.05.2017).
60. Polyvinyl alcohol; MSDS Product No. 341584; Available from Sigma Aldrich.http://www.sigmaaldrich.com/MSDS/MSDS/DisplayMSDSPage.do?country=IN&language=en&productNumber=341584&brand=ALDRICH&PageToGoToURL=%252Fcatalog%252Fproduct%252Faldrich%252F341584%253Flang%253Den(Accessed on 31.05.2017).
61. Melamine; MSDS Product No. M2659; Available from Sigma-Aldrich. http://www.sigmaaldrich.com/MSDS/MSDS/DisplayMSDSPage.do?country=IN&language=en&productNumber=M2659&brand=ALDRICH&PageToGoToURL=http%3A%2F%2Fwww.sigmaaldrich.com%2Fcatalog%2Fsearch%3Fterm%3DMelamine%26interface%3DProduct%2520Name%26N%3D0%2B%26mode%3Dmode%2520matchpartialmax%26lang%3Den%26region%3DIN%26focus%3DproductN%3D0%2520220003048%2520219853286%2520219853147.(Accessed on 27.04.2017).
62. Anthony Kai-chingHau, Tze Hoi Kwan, and Philip Kam-tao Li. Melamine Toxicity and the Kidney. J Am SocNephrol 20: 245–250, 2009.
63. Sodium dodecyl sulfate ; MSDS Product No.L3771; Available from Sigma-Aldrich. http://www.sigmaaldrich.com/MSDS/MSDS/DisplayMSDSPage.do?country=IN&language=en&productNumber=L3771&brand=SIGMA&PageToGoToURL=http%3A%2F%2F

www.sigmaaldrich.com%2Fcatalog%2Fsearch%3Fterm%3DSodium%2Bdodecyl%2Bsulfate%26interface%3DProduct%2520Name%26N%3D0%2B%26mode%3Dmode%2520matchpartialmax%26lang%3Den%26region%3DIN%26focus%3DproductN%3D0%2520220003048%2520219853286%2520219853147 (Accessed on 27.04.2017).
64. Bondi CA, Marks JL, Wroblewski LB, Raatikainen HS, Lenox SR, Gebhardt KE. Human and Environmental Toxicity of Sodium Lauryl Sulfate (SLS): Evidence for Safe Use in Household Cleaning Products. Environ Health Insights. 2015 Nov 17;9:27-32.
65. Sodium ethoxide; MSDS Product No.156248; Available from Sigma-Aldrich. http://www.sigmaaldrich.com/MSDS/MSDS/DisplayMSDSPage.do?country=IN&language=en&productNumber=156248&brand=SIAL&PageToGoToURL=http%3A%2F%2Fwww.sigmaaldrich.com%2Fcatalog%2Fsearch%3Fterm%3DSodium%2Bethoxide%26interface%3DProduct%2520Name%26N%3D0%2B%26mode%3Dmode%2520matchpartialmax%26lang%3Den%26region%3DIN%26focus%3DproductN%3D0%2520220003048%2520219853286%2520219853147 (Accessed on 27.04.2017).
66. https://pubchem.ncbi.nlm.nih.gov/compound/Sodium_ethoxide#section=Chemical-Vendors(Accessed on 02.05.2017).
67. Potassium; MSDS Product No.244864; Available from Sigma-Aldrich. http://www.sigmaaldrich.com/MSDS/MSDS/DisplayMSDSPage.do?country=IN&language=en&productNumber=244864&brand=ALDRICH&PageToGoToURL=http%3A%2F%2Fwww.sigmaaldrich.com%2Fcatalog%2Fsearch%3Fterm%3Dpotassium%26interface%3DAll%26N%3D0%26mode%3Dmatch%2520partialmax%26lang%3Den%26region%3DIN%26focus%3Dproduct (Accessed on 27.04.2017).
68. https://toxnet.nlm.nih.gov/cgi-bin/sis/search/a?dbs+hsdb:@term+@DOCNO+7418 (Accessed on 02.05.2017).
69. Iron(II) chloride; MSDS Product No.372870; Available from Sigma-Aldrich http://www.sigmaaldrich.com/MSDS/MSDS/DisplayMSDSPage.do?country=IN&language=en&productNumber=372870&brand=ALDRICH&PageToGoToURL=http%3A%2F%2Fwww.sigmaaldrich.com%2Fcatalog%2Fsearch%3Fterm%3DIron%2528II%2529%2Bchloride%26interface%3DProduct%2520Name%26N%3D0%2B%26mode%3Dmode%2520matchpartialmax%26lang%3Den%26region%3DIN%26focus%3DproductN%3D0%2520220003048%2520219853286%2520219853147 (Accessed on 27.04.2017).
70. http://www.bioeng.nus.edu.sg/cellular/msds/H2O2.pdf (Accessed on 02.05.2017).
71. Ammonia; MSDS Product No.09684; Available from Sigma-Aldrich. http://www.sigmaaldrich.com/MSDS/MSDS/DisplayMSDSPage.do?country=IN&language=en&productNumber=09684&brand=SIAL&PageToGoToURL=http%3A%2F%2Fwww.sigmaaldrich.com%2Fcatalog%2Fsearch%3Fterm%3Dammonia%26interface%3DAll%26N%3D0%26mode%3Dmatch%2520partialmax%26lang%3Den%26region%3DIN%26focus%3Dproduct (Accessed on 27.04.2017).
72. Environment Canada; Tech Info for Problem Spills: Ammonia (Draft) p.101 (1981).
73. Environment Canada; Tech Info for Problem Spills: Ammonia (Draft) p.103 (1981)
74. Lewis, R.J. Sr. (ed) Sax's Dangerous Properties of Industrial Materials. 11th Edition. Wiley-Interscience, Wiley & Sons, Inc. Hoboken, NJ. 2004., p. 228.
75. Gosselin, R.E., R.P. Smith, H.C. Hodge. Clinical Toxicology of Commercial Products. 5th ed. Baltimore: Williams and Wilkins, 1984., p. III-22.
76. https://toxnet.nlm.nih.gov/cgi-bin/sis/search/a?dbs+hsdb:@term+@DOCNO+162 (Accessed on 28.04.2017).

77. https://toxnet.nlm.nih.gov/cgi-bin/sis/search/a?dbs+hsdb:@term+@DOCNO+909 (Accessed on 02.05.2017).
78. Budavari, S. (ed.). The Merck Index - Encyclopedia of Chemicals, Drugs and Biologicals. Rahway, NJ: Merck and Co., Inc., 1989., p. 334 .
79. Isopropanol; MSDS Product No.W292907; Available from Sigma-Aldrich. http://www.sigmaaldrich.com/MSDS/MSDS/DisplayMSDSPage.do?country=IN&language=en&productNumber=W292907&brand=ALDRICH&PageToGoToURL=http%3A%2F%2Fwww.sigmaaldrich.com%2Fcatalog%2Fsearch%3Fterm%3DIsopropanol%26interface%3DAll%26N%3D0%26mode%3Dmatch%2520partialmax%26lang%3Den%26region%3DIN%26focus%3Dproduct (Accessed on 27.04.2017).
80. Organization for Economic Cooperation and Development; Screening Information Data Set for Sodium Isopropanol (67-63-0) p.21 (January 1998). Available from, as of September 22, 2011: http://www.inchem.org/pages/sids.html
81. N-Methyl-2-pyrrolidone; MSDS Product No.W292907; Available from Sigma-Aldrich.http://www.sigmaaldrich.com/MSDS/MSDS/DisplayMSDSPage.do?country=IN&language=en&productNumber=328634&brand=SIAL&PageToGoToURL=http%3A%2F%2Fwww.sigmaaldrich.com%2Fcatalog%2Fsearch%3Fterm%3DN-Methyl-2-pyrrolidone%26interface%3DAll%26N%3D0%26mode%3Dmatch%2520partialmax%26lang%3Den%26region%3DIN%26focus%3Dproduct (Accessed on 27.04.2017).
82. Malley LA et al; Drug ChemToxicol 24 (4): 315-38 (2001).
83. Gum arabic; MSDS Product No.G9752 ; Available from Sigma-Aldrich. http://www.sigmaaldrich.com/MSDS/MSDS/DisplayMSDSPage.do?country=IN&language=en&productNumber=G9752&brand=SIGMA&PageToGoToURL=http%3A%2F%2Fwww.sigmaaldrich.com%2Fcatalog%2Fsearch%3Fterm%3DGum%2Barabic%2Bfrom%2Bacacia%2Btree%26interface%3DProduct%2520Name%26N%3D0%2B%26mode%3Dmode%2520matchpartialmax%26lang%3Den%26region%3DIN%26focus%3DproductN%3D0%2520220003048%2520219853286%2520219853147 (Accessed on 27.04.2017).
84. Mamleevank, Bakhtizinagz; Gig TrOkhrZdorovyaRabNeftNeftekhim Prom-sti 9: 110-13 (1976).
85. Budavari, S. (ed.). The Merck Index - Encyclopedia of Chemicals, Drugs and Biologicals. Rahway, NJ: Merck and Co., Inc., 1989., p. 334.
86. Cyclohexane; MSDS Product No.P6001; Available from Sigma-Aldrich. http://www.sigmaaldrich.com/MSDS/MSDS/DisplayMSDSPage.do?country=IN&language=en&productNumber=227048&brand=SIAL&PageToGoToURL=http%3A%2F%2Fwww.sigmaaldrich.com%2Fcatalog%2Fsearch%3Fterm%3DCyclohexane%26interface%3DAll%26N%3D0%26mode%3Dmatch%2520partialmax%26lang%3Den%26region%3DIN%26focus%3Dproduct (Accessed on 27.04.2017).
87. Rumack BH POISINDEX(R) Information System Micromedex, Inc., Englewood, CO, 2017; CCIS Volume 172, edition expires May, 2017. Hall AH &Rumack BH (Eds): TOMES(R) Information System Micromedex, Inc., Englewood, CO, 2017; CCIS Volume 172, edition expires May, 2017
88. Sodium dodecylbenzenesulfonate; MSDS Product No. 289957; Available from Sigma-Aldrich. http://www.sigmaaldrich.com/MSDS/MSDS/DisplayMSDSPage.do?country=IN&language=en&productNumber=289957&brand=ALDRICH&PageToGoToURL=http%3A%2F

%2Fwww.sigmaaldrich.com%2Fcatalog%2Fsearch%3Fterm%3DSodium%2Bdodecylbenzenesulfonate%26interface%3DProduct%2520Name%26N%3D0%2B%26mode%3Dmode%2520matchpartialmax%26lang%3Den%26region%3DIN%26focus%3DproductN%3D0%2520220003048%2520219853286%2520219853147 (Accessed on 27.04.2017).
89. n-Vinylcaprolactam; P MSDS Product No.M-218; Scientific polymer Products, Inc. http://scientificpolymer.com/wp-content/uploads/2013/12/M-218-GHS.pdf (Accessed on 02.05.2017).
90. n-Vinylcaprolactam; MSDS Product No.415464 ; Available from Sigma-Aldrich. http://www.sigmaaldrich.com/MSDS/MSDS/DisplayMSDSPage.do?country=IN&language=en&productNumber=415464&brand=ALDRICH&PageToGoToURL=http%3A%2F%2Fwww.sigmaaldrich.com%2Fcatalog%2Fsearch%3Fterm%3Dvinylcaprolactam%26interface%3DAll%26N%3D0%26mode%3Dmatch%2520partialmax%26lang%3Den%26region%3DIN%26focus%3Dproduct (Accessed on 27.04.2017).
91. https://pubchem.ncbi.nlm.nih.gov/compound/1-Hexyl-3-methylimidazolium_hexafluorophosphate#section=Top (Accessed on 02.05.2017).
92. Sodium chlorate; MSDS Product No. SLS4057; Available from ScienceLab.com. http://www.sciencelab.com/msds.php?msdsId=9927592(Accessed on 02.05.2017).
93. Organization for Economic Cooperation and Development; Screening Information Data Set for 1,2-Dichlorobenzene, 95-50-1 p.9 (November 6-9, 2001). Available from, as of January 31, 2008: http://www.chem.unep.ch/irptc/sids/OECDSIDS/sidspub.html.
94. Organization for Economic Cooperation and Development; Screening Information Data Set for 1,2-Dichlorobenzene, 95-50-1 p.106 (November 6-9, 2001). Available from, as of February 4, 2008: http://www.chem.unep.ch/irptc/sids/OECDSIDS/sidspub.html
95. Organization for Economic Cooperation and Development; Screening Information Data Set for 1,2-Dichlorobenzene, 95-50-1 p.179 (November 6-9, 2001). Available from, as of February 7, 2008: http://www.chem.unep.ch/irptc/sids/OECDSIDS/sidspub.html
96. Lewis, R.J. Sr. (ed) Sax's Dangerous Properties of Industrial Materials. 11th Edition. Wiley-Interscience, Wiley & Sons, Inc. Hoboken, NJ. 2004., p. 2920
97. European Chemicals Bureau; IUCLID Dataset, phenyl isocyanate (103-71-9) (2000 CD-ROM edition). Available from, as of March 16, 2012: http://esis.jrc.ec.europa.eu
98. N,N-dimethylformamide; http://nj.gov/health/eoh/rtkweb/documents/fs/1027.pdf
99. N,N-dimethylformamide; MSDS Product No.227056; Available from Sigma-Aldrich. http://www.sigmaaldrich.com/MSDS/MSDS/DisplayMSDSPage.do?country=IN&language=en&productNumber=227056&brand=SIAL&PageToGoToURL=http%3A%2F%2Fwww.sigmaaldrich.com%2Fcatalog%2Fsearch%3Fterm%3DDimethylformamide%26interface%3DProduct%2520Name%26N%3D0%2B%26mode%3Dmode%2520matchpartialmax%26lang%3Den%26region%3DIN%26focus%3DproductN%3D0%2520220003048%2520219853286%2520219853147 (Accessed on 27.04.2017).
100. European Chemicals Bureau; IUCLID Dataset, ACETONE (67-64-1). Available from, as of January 22, 2007: http://esis.jrc.ec.europa.eu/
101. European Chemicals Bureau; IUCLID Dataset, ACETONE (CAS No 67-64-1). Available from, as of Janaury 22, 2007: http://esis.jrc.ec.europa.eu/
102. Verschueren, K. Handbook of Environmental Data of Organic Chemicals. 2nd ed. New York, NY: Van Nostrand Reinhold Co., 1983., p. 150
103. Hexadecyltrimethylammoniumbromide; MSDS Product No.H9151; Available from Sigma Aldrich.

http://www.sigmaaldrich.com/MSDS/MSDS/DisplayMSDSPage.do?country=IN&langua ge=en&productNumber=H9151&brand=SIGMA&PageToGoToURL=http%3A%2F%2F www.sigmaaldrich.com%2Fcatalog%2Fsearch%3Fterm%3DHEXADECYLTRIMETHY LAMMONIUM%2BBROMIDE%26interface%3DAll%26N%3D0%26mode%3Dmatch %2520partialmax%26lang%3Den%26region%3DIN%26focus%3Dproduct (Accessed on 27.04.2017).

104. Sodium dodecyl sulfate; MSDS Product No.71727; Available from Sigma Aldrich. http://www.sigmaaldrich.com/MSDS/MSDS/DisplayMSDSPage.do?country=IN&langua ge=en&productNumber=71727&brand=SIAL&PageToGoToURL=%252Fcatalog%252F product%252Fsial%252F71727%253Flang%253Den (Accessed on 26.05.2017).
105. Dodecylbenzenesulfonic acid; MSDS Product No. 71727; Available from ScienceLab.com. http://www.sciencelab.com/msds.php?msdsId=9923881(Accessed on 26.05.2017).
106. Hexadecyltrimethylammonium bromide; MSDS Product No.H5882; Available from Sigma Aldrich. http://www.sigmaaldrich.com/MSDS/MSDS/DisplayMSDSPage.do?country=IN&langua ge=en&productNumber=H5882&brand=SIGMA&PageToGoToURL=%252Fcatalog%2 52Fproduct%252Fsigma%252Fh5882%253Flang%253Den(Accessed on 26.05.2017).
107. Myristyltrimethylammonium bromide; MSDS Product No. T4762; Available from Sigma Aldrich.http://www.sigmaaldrich.com/MSDS/MSDS/DisplayMSDSPage.do?country=IN &language=en&productNumber=T4762&brand=SIGMA&PageToGoToURL=%252Fcat alog%252Fproduct%252Fsigma%252Ft4762%253Flang%253Den(Accessed on 26.05.2017).
108. Sodium deoxycholate; MSDS Product No.D6750; Available from Sigma Aldrich.http://www.sigmaaldrich.com/MSDS/MSDS/DisplayMSDSPage.do?country=IN &language=en&productNumber=D6750&brand=SIAL&PageToGoToURL=%252Fcatal og%252Fproduct%252Fsial%252Fd6750%253Flang%253Den(Accessed on 30.05.2017).
109. IGEPAL CO-890; MSDS Product No.238678; Available from Sigma Aldrich.http://www.sigmaaldrich.com/MSDS/MSDS/DisplayMSDSPage.do?country=IN &language=en&productNumber=238678&brand=ALDRICH&PageToGoToURL=%252 Fcatalog%252Fproduct%252Faldrich%252F238678%253Flang%253Den(Accessed on 30.05.2017).
110. Tween 20 ; MSDS Product No.P1379; Available from Sigma Aldrich.http://www.sigmaaldrich.com/MSDS/MSDS/DisplayMSDSPage.do?country=IN &language=en&productNumber=P1379&brand=SIAL&PageToGoToURL=%252Fcatalo g%252Fsearch%253Fterm%253DTWEEN%25C2%25AE%2B20%2526interface%253D Product%2BName%2526N%253D0%2526mode%253Dpartialmax%2526lang%253Den %2526region%253DIN%2526focus%253Dproduct (Accessed on 30.05.2017).
111. Tween 80; MSDS Product No.P1754; Available from Sigma Aldrich. http://www.sigmaaldrich.com/MSDS/MSDS/DisplayMSDSPage.do?country=IN&langua ge=en&productNumber=P1754&brand=SIAL&PageToGoToURL=%252Fcatalog%252F search%253Fterm%253Dtween%2B80%2526interface%253DAll%2526N%253D0%252 6mode%253Dpartialmax%2526lang%253Den%2526region%253DIN%2526focus%253 Dproduct(Accessed on 30.05.2017).
112. Tetra(ethylene glycol)diacrylate; MSDS Product No.398802; Available from Sigma Aldrich.http://www.sigmaaldrich.com/MSDS/MSDS/DisplayMSDSPage.do?country=IN

&language=en&productNumber=398802&brand=ALDRICH&PageToGoToURL=%252Fcatalog%252Fproduct%252Faldrich%252F398802%253Flang%253Den(Accessed on 26.05.2017).
113. Triethoxyphenylsilane; MSDS Product No.175609; Available from Sigma Aldrich.http://www.sigmaaldrich.com/MSDS/MSDS/DisplayMSDSPage.do?country=IN&language=en&productNumber=175609&brand=ALDRICH&PageToGoToURL=%252Fcatalog%252Fproduct%252Faldrich%252F175609%253Flang%253Den(Accessed on 26.05.2017).
114. 3-Glycidyloxypropyl)trimethoxysilane; MSDS Product No.440167; Available from Sigma Aldrich.http://www.sigmaaldrich.com/MSDS/MSDS/DisplayMSDSPage.do?country=IN&language=en&productNumber=440167&brand=ALDRICH&PageToGoToURL=%252Fcatalog%252Fproduct%252Faldrich%252F440167%253Flang%253Den253Den(Accessed on 26.05.2017).
115. Iron(III) chloride; MSDS Product No.F7134; Available from Sigma Aldrich.http://www.sigmaaldrich.com/MSDS/MSDS/DisplayMSDSPage.do?country=IN&language=en&productNumber=F7134&brand=SIAL&PageToGoToURL=%252Fcatalog%252Fproduct%252Fsial%252Ff7134%253Flang%253Den(Accessed on 26.05.2017).
116. Nitromethane; MSDS Product No.108170; Available from Sigma Aldrich.http://www.sigmaaldrich.com/MSDS/MSDS/DisplayMSDSPage.do?country=IN&language=en&productNumber=108170&brand=SIAL&PageToGoToURL=%252Fcatalog%252Fproduct%252Fsial%252F108170%253Flang%253Den(Accessed on 26.05.2017).
117. 1-butyl-3-methyl-imidazoliumbis(trifluoromethanesulfonyl)imide; MSDS Product No.77896; Available from Sigma Aldrich.http://www.sigmaaldrich.com/MSDS/MSDS/DisplayMSDSPage.do?country=IN&language=en&productNumber=77896&brand=ALDRICH&PageToGoToURL=%252Fcatalog%252Fproduct%252Faldrich%252F77896%253Flang%253Den(Accessed on 30.05.2017).
118. 1,2-dichloroethane; MSDS Product No.284505; Available from Sigma Aldrich.http://www.sigmaaldrich.com/MSDS/MSDS/DisplayMSDSPage.do?country=IN&language=en&productNumber=284505&brand=SIAL&PageToGoToURL=%252Fcatalog%252Fproduct%252Fsial%252F284505%253Flang%253Den253Den(Accessed on 31.05.2017).
119. Propylene carbonate; MSDS Product No.310328; Available from Sigma Aldrich.http://www.sigmaaldrich.com/MSDS/MSDS/DisplayMSDSPage.do?country=IN&language=en&productNumber=310328&brand=SIAL&PageToGoToURL=%252Fcatalog%252Fproduct%252Fsial%252F310328%253Flang%253Den(Accessed on 31.05.2017).
120. Sulfuric acid; MSDS Product No.435589; Available from Sigma Aldrich.http://www.sigmaaldrich.com/MSDS/MSDS/DisplayMSDSPage.do?country=IN&language=en&productNumber=435589&brand=SIAL&PageToGoToURL=%252Fcatalog%252Fproduct%252Fsial%252F435589%253Flang%253Den(Accessed on 31.05.2017).
121. Potassium chlorate; MSDS Product No.255572; Available from Sigma Aldrich.http://www.sigmaaldrich.com/MSDS/MSDS/DisplayMSDSPage.do?country=IN

&language=en&productNumber=255572&brand=SIGALD&PageToGoToURL=%252Fcatalog%252Fsearch%253Fterm%253DKClO3%2526interface%253DAll%2526N%253D0%2526mode%253Dpartialmax%2526lang%253Den%2526region%253DIN%2526focus%253Dproduct(Accessed on 31.05.2017).

122. Sodium nitrate; MSDS Product No. S5506; Available from Sigma Aldrich.http://www.sigmaaldrich.com/MSDS/MSDS/DisplayMSDSPage.do?country=IN&language=en&productNumber=S5506&brand=SIGALD&PageToGoToURL=%252Fcatalog%252Fsearch%253Fterm%253DNaNO3%2526interface%253DAll%2526N%253D0%2526mode%253Dpartialmax%2526lang%253Den%2526region%253DIN%2526focus%253Dproduct(Accessed on 31.05.2017).

123. Potassium permanganate; MSDS Product No.60458; Available from Sigma Aldrich.http://www.sigmaaldrich.com/MSDS/MSDS/DisplayMSDSPage.do?country=IN&language=en&productNumber=60458&brand=SIGALD&PageToGoToURL=%252Fcatalog%252Fsearch%253Fterm%253DKMnO4%2526interface%253DAll%2526N%253D0%2526mode%253Dpartialmax%2526lang%253Den%2526region%253DIN%2526focus%253Dproduct(Accessed on 31.05.2017).

124. Larson, L.L., Kenaga, E.E., Morgan, R.W. Commercial and Experimental Organic Insecticides. 1985 Revision. College Park, MD: Entomological Society of America, 1985., p. 25.

125. Ruth JH; Am IndHygAssoc J 47: A-142-51 (1986)

126. Chloroform solution; MSDS Product No.487163 ; Available from Sigma-Aldrich. http://www.sigmaaldrich.com/MSDS/MSDS/DisplayMSDSPage.do?country=IN&language=en&productNumber=487163&brand=SIAL&PageToGoToURL=http%3A%2F%2Fwww.sigmaaldrich.com%2Fcatalog%2Fsearch%3Fterm%3DChloroform%26interface%3DAll%26N%3D0%26mode%3Dmatch%2520partialmax%26lang%3Den%26region%3DIN%26focus%3Dproduct (Accessed on 27.04.2017).

127. (a) Jeon, I-Y,. et al.Large-Scale Production of Edge-Selectively Functionalized GrapheneNanoplatelets via Ball Milling and Their Use as Metal-Free Electrocatalysts for Oxygen Reduction Reaction. J. Am. Chem. Soc. **135**, 1386–1393, (2013).(b) Jeon, I-Y,.etal.Scalable Production of Edge-Functionalized GrapheneNanoplatelets via Mechanochemical Ball-Milling.Adv. Funct. Mater. **135**, 6861-6975, (2015). (c) Jeon, I-Y,.etal.Edge-carboxylatedgraphenenanosheets via ball milling. PNAS**109**, 5588–5593, (2012)

128. Sulfur trioxide; MSDS Product No.227692; Available from Sigma-Aldrich.http://www.sigmaaldrich.com/MSDS/MSDS/DisplayMSDSPage.do?country=IN&language=en&productNumber=227692&brand=ALDRICH&PageToGoToURL=%252Fcatalog%252Fproduct%252Faldrich%252F227692%253Flang%253Den(Accessed on 03.06.2017).

129. H, J, Salavagione., J, Sherwood., M, Debruyn., V, L, Budarin., G, J, Ellis.,J, H, Clark.& P, S, Shuttleworth. Identification of high performance solvents for thesustainableprocessing of graphene. *Green Chemistry***19,** 2550-2560, (2017).

130. Cyrene; MSDS Product No.807796; Available from Sigma-Aldrich.http://www.sigmaaldrich.com/MSDS/MSDS/DisplayMSDSPage.do?country=IN&language=en&productNumber=807796&brand=SIAL&PageToGoToURL=http%3A%

2F%2Fwww.sigmaaldrich.com%2Fcatalog%2Fproduct%2Fsial%2F807796%3Flang%3Den (Accessed on 19.06.2017).

131. R. Jangle, B. Thorat, Reversed-phase high-performance liquid chromatography method for analysis of curcuminoids and curcuminoid-loaded liposome formulation, Indian J. Pharm. Sci. 75 (2013) 60–66.
